# Insensitivity of Place Cells to the Value of Spatial Goals in a Two-Choice Flexible Navigation Task

**DOI:** 10.1523/JNEUROSCI.1578-18.2018

**Published:** 2019-03-27

**Authors:** Éléonore Duvelle, Roddy M. Grieves, Vincent Hok, Bruno Poucet, Angelo Arleo, Kate J. Jeffery, Etienne Save

**Affiliations:** ^1^Aix Marseille University, Centre National de la Recherche Scientifique (CNRS), Laboratory of Cognitive Neuroscience, Marseille, France,; ^2^Sorbonne Université, INSERM, CNRS, Institut de la Vision, F-75012 Paris, France, and; ^3^Institute of Behavioural Neuroscience, Division of Psychology and Language Sciences, University College London, London WC1H 0AP, United Kingdom

**Keywords:** goal value, goal-directed behavior, hippocampus, place cells, rat

## Abstract

Hippocampal place cells show position-specific activity thought to reflect a self-localization signal. Several reports also point to some form of goal encoding by place cells. We investigated this by asking whether they also encode the value of spatial goals, which is crucial information for optimizing goal-directed navigation. We used a continuous place navigation task in which male rats navigate to one of two (freely chosen) unmarked locations and wait, triggering the release of reward, which is then located and consumed elsewhere. This allows sampling of place fields and dissociates spatial goal from reward consumption. The two goals varied in the amount of reward provided, allowing assessment of whether the rats factored goal value into their navigational choice and of possible neural correlates of this value. Rats successfully learned the task, indicating goal localization, and they preferred higher-value goals, indicating processing of goal value. Replicating previous findings, there was goal-related activity in the out-of-field firing of CA1 place cells, with a ramping-up of firing rate during the waiting period, but no general overrepresentation of goals by place fields, an observation that we extended to CA3 place cells. Importantly, place cells were not modulated by goal value. This suggests that dorsal hippocampal place cells encode space independently of its associated value despite the effect of that value on spatial behavior. Our findings are consistent with a model of place cells in which they provide a spontaneously constructed value-free spatial representation rather than encoding other navigationally relevant but nonspatial information.

**SIGNIFICANCE STATEMENT** We investigated whether hippocampal place cells, which compute a self-localization signal, also encode the relative value of places, which is essential information for optimal navigation. When choosing between two spatial goals of different value, rats preferred the higher-value goal. We saw out-of-field goal firing in place cells, replicating previous observations that the cells are influenced by the goal, but their activity was not modulated by the value of these goals. Our results suggest that place cells do not encode all of the navigationally relevant aspects of a place, but instead form a value-free “map” that links to such aspects in other parts of the brain.

## Introduction

Goal-directed navigation mobilizes a large network of brain areas, central to which is the hippocampus. In mammals, the dorsal hippocampal CA1 and CA3 regions contain place cells, the firing of which is localized to “place fields” and encodes an animal's position in an environment (O'Keefe and Dostrovsky, 1971; Moser et al., 2008). The dorsal hippocampus is important for place navigation (Morris et al., 1982; Moser et al., 1995), and so knowing whether place cells encode information about spatial goals is fundamental to understanding navigation mechanisms. The focus of the present study is whether the values of spatial goals are encoded by the hippocampus.

Evidence that hippocampal neurons may encode goal locations differently from neutral places is mixed (Poucet and Hok, 2017). Some studies have reported no goal responsiveness (Speakman and O'Keefe, 1990; Zinyuk et al., 2000; Jeffery et al., 2003; Grieves et al., 2016; Spiers et al., 2018), whereas others have found increased activity during goal approach (Eichenbaum et al., 1987; Wiener, 1993; Breese et al., 1989). Recent studies have observed an increased population firing at the goal, occurring away from the place field location (Hok et al., 2007a,b, 2013; Hayashi et al., 2016). Finally, other studies have shown that place fields migrate toward, or overrepresent, goal locations (Hollup et al., 2001; Kobayashi et al., 2003; Lee et al., 2006; Dupret et al., 2010; McKenzie et al., 2013; Tryon et al., 2017). Collectively, these studies suggest that the occurrence of goal coding depends on a conjunction of factors such as task demands, intertrial continuity, goal novelty, or trajectory stereotypy (repeated traversals of the same path).

Many navigational decisions require choosing the best among multiple goals, but few studies have investigated the neural representation of goal *value* in the hippocampus. One such study found no evidence of hippocampal encoding of goal value (Tabuchi et al., 2003), but the spatial demands of this task were low. Others have suggested that place cells may encode reward probability, action value, or reward expectation (H Lee et al., 2012; Lee et al., 2017; Tryon et al., 2017) in linear mazes with no need for localizing a hidden goal. The amount of reward available at a goal seems to affect some hippocampal phenomena such as sharp-wave ripples in the local field potential (Singer and Frank, 2009) or patterns of sequential place cell activation (“replay”; Ambrose et al., 2016), but these events happen at the time of reward consumption and might reflect a reward-related feedback signal rather than a representation of goal value. Therefore, the question of whether place cells encode the value of spatial goals is still open.

To address this question, we modified a task we have previously used to investigate hippocampal goal coding (Hok et al., 2007a). The continuous navigation task (adapted from Rossier et al., 2000) requires animals to navigate to an unmarked location in an open field and wait there for a short duration (2 s), after which an overhead dispenser releases a food pellet that the animal has to search for. This task dissociates goal location from reward consumption, and allows recording of place fields because the animal covers the whole environment during its search for the reward. We previously found that CA1 place cells with place fields located away from the goal fire spikes when the animal waits in the goal zone (Hok et al., 2007a), suggesting possible goal encoding. The task that we designed has two simultaneous goals that could provide different amounts of food, thus adding a value-based decision-making component to this spatial task.

We found that rats were able to locate the two goals and preferentially navigate to the higher-value goal, indicating behavioral sensitivity to this parameter. However, we did not observe any place field overrepresentation of the goals and saw no evidence of consistent goal value coding by place cells. We conclude that place cells do not encode the value of spatial goals and that, instead, this information must be combined with place information outside of the hippocampus.

## Materials and Methods

### Subjects

Six male Long–Evans rats (Janvier Labs) weighing 230–250 g and aged ∼2 months at the start of the experiment were used. Upon arrival, they were housed two per cage in a colony room at 20 ± 2°C under a 12 h/12 h light/dark cycle beginning at 7:00 A.M. with *ad libitum* access to food and water. They were handled daily for 10 d. Before behavioral training began, animals underwent a food deprivation procedure until they reached 90% of free-feeding body weight, and were maintained between 90% and 95% of the free-feeding weight during the study. After implantation surgery they were housed individually. The procedures were approved by the local ethics committee (authorization #A81212) and the experiments were performed in accordance with European (2010/63/UE) and French (Council Directive 87848 and permission #13.24 to E.S.) guidelines.

### Behavioral apparatus

**Figure 1.**
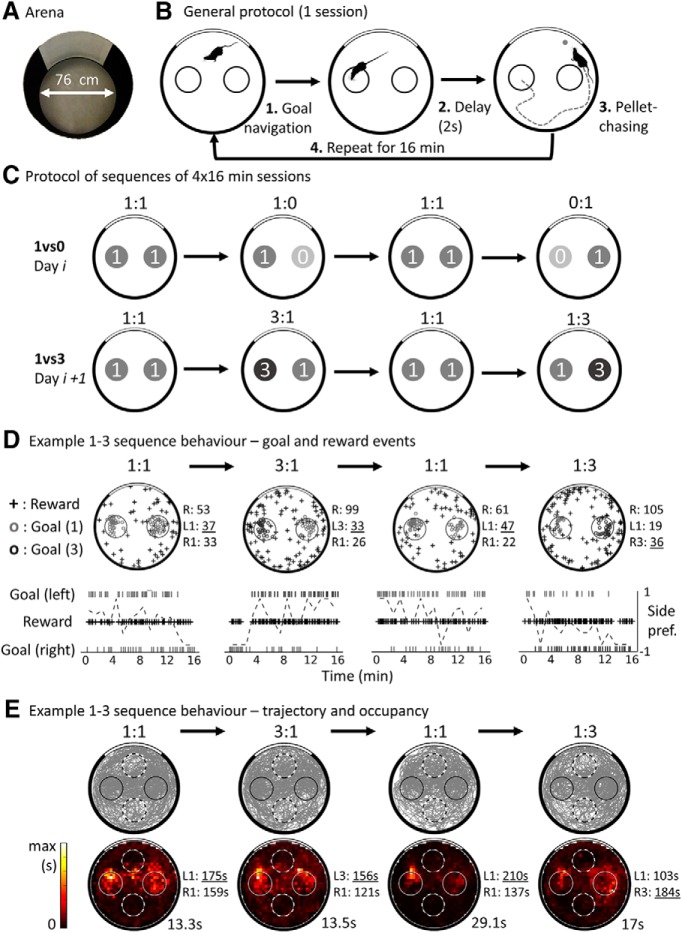
Protocol and behavior in the two-goal navigation task. ***A***, View of the task arena from above. ***B***, Task protocol. First, the rat navigates to one of the two goal zones, represented as small circles and corresponding to 20-cm-diameter unmarked zones located to the left or right of a white cue card. If the rat waits for 2 s in a goal, then a food pellet is released from an overhead dispenser and stops at a random location. Then, the rat leaves the goal to find and consume the food. The process repeats during 16 min sessions. ***C***, Example of two possible sequences of sessions; a specific sequence was performed on a given day, with no indication of change between its sessions. A control session was always performed first (equal rewards, 1:1), followed by a session with different values (e.g., 1:0, right goal unrewarded), then another control session (1:1), and finally the mirrored version of the first value session (e.g., 0:1, left goal unrewarded). On the next day, a sequence with the other set of goal values was usually performed (in 1:3 or 3:1, the right or left goal provides 3 simultaneous pellets). ***D***, Top, location of events from an example 1vs3 sequence. Reward consumption events are shown as crosses; correct (duration ≥ 2 s) goal visit events are shown as small circles. The number of recorded events of each type is shown at the right of each plot; the highest number of goal visits is underlined for each session. Note that reward locations appear randomly distributed in the environment and that goal events are concentrated on the goal zones (with rare occurrences of mistracked events). Bottom, Timing of events from the same 1:3 sequence: raster marks at the top and bottom show left and right goal activation events, respectively. Cross marks show reward consumption events. The dotted line shows the side preference index of the rat toward the left or right (see Materials and Methods) computed in 1 min time bins. Note that the preference is approximately balanced in the first session, but switches toward the high-value goal on the 3:1 and 1:3 sessions, indicating sensitivity of the rat to the changing reward values. ***E***, Rat trajectory (top) and occupancy map (bottom) for the example sessions shown in ***D***. The gray lines show the path of the rat; goals are shown as solid circles and control nongoal zones as dotted circles. The peak time for each occupancy map is shown in seconds.

Training and electrophysiological recordings were performed in a 76-cm-diameter circular arena (see Fig. 1*A*) with 50-cm-high black metallic walls and a gray-painted wooden floor. A white cue covering 100° of arc was painted on the wall from top to bottom (making it 50 cm high). The arena was located in the middle of a circular enclosure of opaque black curtains 2.5 cm diameter and 2.5 m high. A food dispenser (Med Associates) was located 2 m above the arena floor. When the dispenser was activated, one or more (depending on the experimental condition) 20 mg food pellets (A/P formula; TestDiet) dropped into the arena below. Pellets were released randomly through four angled exit tubes and would roll to an unpredictable location in the arena (see Fig. 1*D* for example reward locations). Because the animal had to forage over the entire arena to retrieve the pellet(s), good sampling of all locations was obtained during recordings. Two cameras were located above the arena, one allowing tracking of the animal's head position and the other allowing visual detection of the pellet consumption (see details below). A radio set tuned to an FM broadcast station and located above the apparatus was used to try to mask incidental auditory cues. The apparatus was indirectly lighted by four symmetrically positioned LED spots. The recording setup and computers for experimental control were located in an adjacent room. The experimenter entered the recording room at the start and the end of a sequence of sessions, and occasionally otherwise to clean urine traces or disentangle the recording cable.

### Training procedure

The rats were trained in an adapted version of the continuous navigation task used by Hok et al. (2007a), hereafter called the two-goal navigation task. In that previous study, the animals were trained to locate a non-cued circular goal zone and stay there for at least 2 s, at which time an overhead food dispenser was activated to release a 20 mg pellet. In the present study, a similar procedure was used (Fig. 1*B*) except that the animals could visit two symmetrically placed non-cued goal zones (each 20 cm diameter). Staying in either of the two goal zones for 2 s triggered the dispenser, after which the rat searched for and consumed the pellet, at which point that trial terminated and the next began.

Training was performed in four steps. For each step, two consecutive 16 min training sessions were conducted each day until the animals reached a learning criterion of one visit per goal zone per minute. The rat was left in the arena between sessions and was returned to its home cage at the end of the sequence. The arena floor was then wiped with water to reduce and disperse local olfactory cues. The training steps were as follows.

In step 1, a linoleum disc (20 cm diameter) was placed on the floor, cueing one of the two goal zones during the first session. The dispenser was automatically activated upon detection of the animal in the cued goal zone. In the second session, the disc was removed and the animal was rewarded when it entered the non-cued goal zone. On the following day, the same procedure was repeated for the other goal zone.

In step 2, the animal had to stay in each goal zone for a delay that gradually increased across days in order to automatically activate the dispenser. The delay was increased from 0.5 to 2 s in 0.5 s steps every other day over successive training sessions, using the same goal location. A similar sequence of daily sessions with exposure to each goal zone (marked followed by unmarked sessions) was conducted as in step 1.

In step 3, the two goal zones were simultaneously available during a given session. Each day, a session with the marked goal zones was followed by a session with the unmarked goal zones.

Step 4 was the final form of this task, in which the two unmarked goal zones were simultaneously available during each of the two daily sessions. The reward was released after a 2 s delay spent in a goal zone. To obtain a similar number of visits to each goal zone during a session, a 5 s refractory period (minimum time between two consecutive dispenser activations from the same goal zone) was used. This meant that the rat had to spend >3 s outside of a goal zone before being able to reactivate it. Successful visits to the goal zones were consistently followed by a foraging episode to retrieve the pellet. Once the pellet was found and eaten, the animal returned immediately to a goal zone. At the end of training, animals that had a significant bias toward one of the goals were submitted to a training session where that goal zone was unrewarded followed by a normal session, repeated twice each day until they showed no significant preference for a specific goal.

The overall training phase lasted ∼6 weeks. Only posttraining behavioral data were analyzed.

### Electrode implantation

Following training, animals underwent surgery for electrode implantation above the right dorsal hippocampus. Four rats were implanted above the CA1 field (aiming to collect CA1 followed by CA3 data) and, to eliminate possible experience-dependent effects on results, two rats were implanted directly above the CA3 field (coordinates relative to bregma, AP −3.8 mm, L −3.0 mm, DV to dura −1.5 mm for CA1, −2.5 mm for CA3, Paxinos and Watson, 2007). A drivable bundle of four tetrodes (Kubie, 1984) was implanted surgically under general anesthesia using intraperitoneal ketamine 60 mg/kg (Imalgène 1000; Mérial) and intraperitoneal medetomidine 0.25 mg/kg (Domitor; Janssen). Each tetrode was composed of four twisted 25 μm nichrome wires. The four tetrodes formed a bundle threaded through a length of 30-gauge stainless steel tubing. Each wire was connected to a pin of an 18-pin Mill-Max connector. The tubing was attached to the central pin of the connector and served as the animal ground as well as a guide for the tetrodes. The connector, tubing, and three drive screws were embedded in acrylic to form a triangle. The tetrodes could be lowered in the brain by turning the three drive screws (1 turn ≈ 450 μm) inserted in nylon cuffs cemented to the skull. Before surgery, the wire tips were gold plated to reduce their impedance to 200–400 kΩ (measured at 1 kHz). Presurgery and postsurgery treatments included a long-acting antibiotic (amoxicillin, 150 mg/kg, s.c.) and an analgesic (buprenorphine, 0.05 mg/kg, s.c.). After surgery, the animals were placed in a recovery room (22°C) for 3 d before being returned to the colony room.

### Screening and recording

Following a recovery period of at least 10 d postsurgery, rats were screened daily while performing the two-goal navigation task. If single-unit signals were considered of sufficient amplitude, then the recording protocol began. Otherwise, the electrodes were lowered by ∼28–56 μm and the rat was returned to its home cage. A delay of 24 h was interposed between successive screening sessions.

During screening and recording, a cable connected the recording system via a turning commutator to the headstage containing an operational amplifier (TLC 2272; Texas Instruments) plugged into the rat's microdrive. A pulley and weight system helped to compensate for the weight of the recording cable. Neural activity was recorded using 16-channel Neuralynx hardware controlled by a SciWorks acquisition system (Datawave). LFPs were sampled continuously from one channel at a rate of 724 Hz (gain 1000, filtered 1–475 Hz). Unit signals were amplified 10,000 times and filtered (0.3 Hz–6 kHz). During recording, waveforms with amplitudes exceeding an experimenter-set threshold were sampled at 32 kHz and stored.

A single red LED was connected at the back of the headstage, allowing monitoring of the animal's head position with a 50 Hz sampling rate by a tracking system (Videotrack; Viewpoint). The tracking system was interfaced with the recording software and the pellet delivery device so that detection of the LED in a goal zone for 2 s automatically triggered an event flag, together with appropriate activation of the reward dispenser. The activation of the dispenser produced a small sound, as did the pellet(s) landing on the arena floor. An additional event flag was manually entered as a key press, the time stamp of which was automatically saved by the recording system, when the animal was seen to eat a pellet by an experimenter watching a screen. In addition to the Videotrack system that was used to detect the animal in the goal zones, a Datawave tracker (50 Hz sampling rate) was used to combine the animal's head position with unit signals.

The recording protocol included one of two types of sequences of four 16 min sessions each (Fig. 1*C*). On a given day, either a 1vs0 or a 1vs3 sequence was performed. The 1vs0 sequence consisted of alternating 1:1 sessions (each successful goal visit released 1 pellet) with 1:0 sessions (one of the goals did not release a pellet). Sequences always started with a 1:1 session and the choice of which goal set to 0 was reversed between the first and second 1:0 sessions. The 1vs3 sequence similarly alternated 1:1 and 1:3 sessions (where one of the goals provided three pellets released at 200 ms intervals). Similarly, the side of the goal providing three pellets was swapped between the first and second 1:3 session. As in training, a goal could not be reactivated during the 5 s following its activation to promote visits of the two goals even in value-changing conditions. Figure 1*D* shows an example of goal activation and pellet consumption behavior in a 1 vs 3 sequence spatially and temporally. Importantly, no exterior signal indicated session change, and the transition between sessions was continuous: rats could only rely on the reward provided by each goal to estimate goal values.

At the end of the first day, the electrodes were left in place to allow the possibility of recording the same neurons in the other sequence on the next day. Once signals were recorded in each sequence, tetrodes were moved 0 to 1/8 of a screw turn (∼0–50 μm).

### Data analysis

#### Position tracking and behavior

All analyses and statistics were performed using the Python Programming Language, apart from data conversion and LFP analyses, which were implemented in MATLAB (The MathWorks).

First, out-of-arena (mistracked) points were removed, and positions were speed filtered such that any point with instantaneous speed >150 cm/s was removed. Missing positions were interpolated and all positions smoothed using a moving average over 9 points. Instantaneous speed was computed on a window of three position data points, then smoothed with a moving average over nine position points. Speed data were used to filter spuriously high-speed tracked positions (see above). Then, speed was recomputed on corrected position data and used to compute speed-filtered occupancy and rate maps. An example of corrected position data for a sequence of sessions is shown in Figure 1*E*.

Color-coded occupancy maps were built to visualize the distribution of the time spent by the rat in various parts of the environment. Position data were binned into 32 × 32 bins (∼2.3 cm^2^) and dwell time in each bin computed to visualize the distribution of rat position (see Fig. 1*E* for examples). We also compared the occupancy in the goal locations with matching control locations of the same size but rotated by 90 degrees. To quantify possible biases in goal choice, we calculated a spatial preference index as follows:

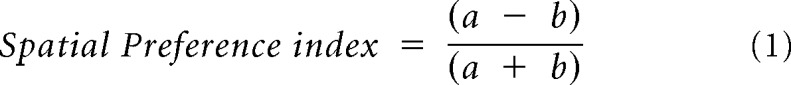
 where *a* = number of correct (longer than 2 s) visits to the left goal zone and *b* = number of correct visits to the right goal zone. Left/right referred to the position of each goal zone with respect to the cue card. To quantify the preference for a goal depending on its value (i.e., amount of reward provided), a value preference index was computed using the same method as spatial preference, but where *a* represented the number of visits to the high value goal zone and *b* the number to the low value goal zone. Spatial and value preference indices were computed either on whole sessions (16 min) to evaluate the global preference or in 1 min bins to analyze the evolution of preference within sessions.

Speed profiles around task events (goal activation and reward consumption) were constructed by computing the average speed profile time-locked on the event (4 s before, 2 s after) for each session, then combining events of the same type (equal-, high-, or low-value events) and averaging over sessions.

#### Single units

##### Spike sorting.

Spike-sorting was performed manually for each session to ensure good quality isolation according to previously published methods (Hok et al., 2007a,b) using the Offline sorter software (Plexon). Putative spiking events were grouped based on waveform properties including waveform shape, peak amplitude, peak-to-valley amplitude, spike amplitude at experimenter-defined times, and spike duration. Clusters with >1% spikes having interspike intervals <2 ms (indicating poor cluster discrimination) were discarded (Alexander et al., 2016; Tanila et al., 2018).

##### Rate maps.

Once putative cells had been isolated several types of firing rate maps were built to visualize and analyze the spatial distribution of firing rate for each recording session. First, each spike was associated to the temporally closest recorded position. The recording arena was divided, as mentioned above, into 32 × 32 square bins and rate maps were computed as the number of spikes per bin divided by the time spent in each bin using only bins visited for >0.1 s. Smoothed firing rate maps were built from these maps using a Gaussian filter of σ 0.7 (using the *scipy.ndimage.Gaussian_filter* function). The same smoothing parameters were applied to all types of rate maps used for the analysis. Speed-filtered firing rate maps (whether smoothed or not) were constructed similarly but using speed-filtered spikes and position (speed > 15 cm/s, Bendor and Wilson, 2012). Smoothed, speed-filtered rate maps were used to detect place fields and compute spatial information content. Smoothed “low-speed” maps (speed <15 cm/s) were used to test for speed-dependent alterations in firing. As will be shown later (in the behavioral results section), the mean speed of rats during the goal activation delay is ∼10 cm/s, which is why we chose the threshold of 15 cm/s to encompass most of the data at the goal within the low-speed condition, as well as data with similar speed elsewhere in the arena. Finally, smoothed “task-phase” maps were built dissociating the reward-chasing phase (accumulation of episodes starting at a goal event and ending at the pellet consumption event preceding the next goal activation) from the goal-directed phase (starting from each pellet consumption event directly preceding a recorded goal visit to the next goal-visit event, delay period included). Only the trials that included a recorded food consumption event were used as some events were missed by the experimenter (∼21 ± 10% on average, computed using the difference between the recorded and theoretical count).

Place fields were defined as a group of at least nine contiguous pixels (sharing a side) with firing rate exceeding 20% of the peak firing rate in the smoothed, speed-filtered (speed >15 cm/s) rate map (Muller et al., 1987; Park et al., 2011; Brandon et al., 2014; Mamad et al., 2017; Tanaka et al., 2018). Several parameters were computed on place fields: mean place field firing rate, peak place field firing rate, and place field size. Place fields and other cell parameters were always defined for each session separately.

Information content (i.e., the amount of information in bits per second conveyed about spatial location by a cell; Skaggs et al., 1993) was calculated according to the following formula:

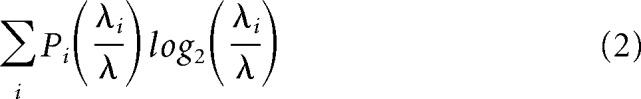
 where λ*_i_* is the mean firing rate in each pixel, λ is the overall mean firing rate, and *P_i_* is the probability of the animal to be in the pixel *i* (i.e., dwell time in pixel/total dwell time). This was computed over all pixels *i* of the smoothed speed-filtered (speed >15 cm/s) rate map.

A burst index was computed on whole-session data as the percentage of interspike intervals shorter than one-fourth of each unit's mean interspike interval (H Lee et al., 2012). The waveform duration was computed on each cluster's representative waveform (i.e., the waveform of highest amplitude between all four channels averaged over all spikes of a 1vs1 session) as the peak-to-trough duration.

All rate maps and spike plots presented here have been rotated to show the cue card at the top of the figure, but analyses were performed on original, unrotated data.

##### Cell classification.

Using waveform and firing characteristics, each cluster (from a given recording session) was automatically classified into a particular cell type. Place cells were classified using the following criteria: burst index >30%, waveform peak to trough duration >300 μs, mean speed-filtered firing rate between 0.05 and 7 Hz, at least 1 place field, and spatial information content >0.5 bits/s. A substantial number of the other pyramidal clusters had very low firing and no place field and these were estimated to be “silent cells”: pyramidal neurons that have the ability to develop a place field under certain conditions (Thompson and Best, 1989; Epsztein et al., 2011; D Lee et al., 2012; Diamantaki et al., 2018). Only place cells were analyzed here.

##### Cell matching.

Once individual clusters were spike-sorted and classified, clusters belonging to the same cell recorded in different sessions had to be identified. This was done in two ways depending on the situation: (1) for sessions recorded on the same day (i.e., without unplugging the rat), clusters were manually associated to the same cell depending on their position in the cluster space; and (2) for sessions recorded on successive days, an automatized procedure comparing the waveforms was used (Tolias et al., 2007, see below), followed by manual refinement. For each tetrode, each cluster recorded on the first session of a given day was compared with all clusters recorded during the first session of the next recording day, when the electrodes had not been moved by more than ∼0.5 screw turn (0–200 μm). The automated procedure was as follows. First, two distance measures (“Tolias distances”) were computed on each pair of averaged waveforms (consisting of the average waveform for each electrode of the tetrode). The first measure captures the difference in waveform shapes, once scaled to the same amplitude, and the second describes the difference in amplitudes across all four electrodes (for more details, see Tolias et al., 2007 and Powell and Redish, 2014). Then, a linear discriminant analysis method was applied that used the Tolias distances to classify pairs of averaged waveforms as “same” or “different” (Powell and Redish, 2014). As a control group of pairs of same waveforms, we used the distances between the “same” units recorded from different sessions of the same day. As a control group of “different” waveforms, we used the distances between units recorded more than ∼200 μm apart. These two groups were fed to a linear discriminant analysis classifier (*sklearn.discriminant_analysis LinearDiscriminantAnalysis* Python function), which was then used to categorize pairs of averaged waveforms into either the “same” or the “different” group, using the two Tolias distances as dimensions. Finally, the averaged waveforms from the two clusters were manually checked by the experimenter using the result of the classifier as well as the waveforms and the speed-filtered rate maps. The automated and manual method agreed on >70% of all matched clusters (the cells belonging to the remaining <30% pairs were considered to be different cells in the rest of the analysis). A similar proportion of cluster pairs that were not matched by the algorithm (<30%) were matched by the experimenter. This final classification was used for all the analyses. Unless stated otherwise, when a given cell parameter was analyzed for a specific condition, the average of this parameter over all instances of this cell in the condition was used.

##### Goal overrepresentation by place fields.

To assess whether place fields overrepresent the goal locations, we computed a goal representation index for each condition, indicating the proportion of fields located at the goals. For cells recorded several times in the same condition, the session with the highest mean firing rate was considered. Two methods were used and each was applied twice: once considering all fields and once considering only one field per place cell (the largest). Both methods relied on defining goal fields and control fields. For the first method, goal fields were place fields with their center-of-mass (COM) located inside any of the goals, and control fields were those with a COM inside any of the control zones equivalent in size to the goals (see Fig. 1*E* for an illustration of the location of the control zones). For the second method, goal fields were those closest to the goals and control fields were those closest to the control zones (therefore, all fields were included in method 2 but only a subset in method 1). For both methods, the goal representation index was equal to the number of goal fields divided by the number of all included fields (goal + control). A goal representation index of 50% would thus indicate equivalent representation of the goals and the control zones. Finally, to assess whether the goal representation index was significantly higher than chance, we computed shuffled goal representation indices by randomly creating the same number of COMs as in the corresponding dataset with their coordinates contained within the boundaries of the recording arena, and recomputing the index from these; 1000 shuffled goal representation indices were created for each condition. If the experimental value was above the 95th percentile of the shuffled distribution, then it indicated overrepresentation of the goals (one-sided test).

A firing rate approach was also used to assess whether the firing of place fields was different at the goals than at the control zones. For this analysis, we compared the firing rate at goal zones of fields encroaching on a goal (i.e., for which any of the pixels of the field was contained in a goal zone) with the firing rate at control zones of fields encroaching on these zones. Therefore, different cells could contribute to each category and some cells might be used both for the goal and control group if they had a large enough field. The two groups of firing rate were then compared using a Mann–Whitney *U* test. To illustrate this analysis, cumulated rate maps were computed by taking one *Z*-scored, speed-filtered, and smoothed rate map per cell and per condition (in the case of multiple recordings of the cell, the rate map with highest average firing rate was used) and averaging over all *Z*-scored maps for each condition. Only bins with data from at least two cells were included and the final maps were smoothed again, as described above.

##### Task phase correlates.

The place cell population might change its activity (or “remap”) between the different subphases of the two-goal navigation task (i.e., goal-directed phase vs pellet-chasing phase, as suggested in Hok et al., 2007a; see also Markus et al., 1995). To evaluate this, we built phase-filtered place cell maps (goal-directed vs reward-searching phases or slow vs high speed phases) and computed the Pearson's *R* correlation coefficient between the two types of maps for each cell. Then, we built a distribution of shuffled correlations between the same phase maps but from different cells recorded in the same session (only including place cells). Therefore, only sessions with at least two simultaneously recorded place cells contributed to the shuffle distributions. Furthermore, to assess whether the distribution of correlations obtained was bimodal, possibly indicating that different subpopulations of place cell would behave differently, we used Hartigan's dip test (Hartigan and Hartigan, 1985, MATLAB implementation).

##### Spatial firing stability.

To assess whether place cells were stable between successive sessions and whether reward-changing sessions would influence this, we computed the Pearson's *R* correlation between smoothed, speed-filtered rate maps from one session to all other sessions recorded for that cell (i.e., also across days).

##### Overdispersion.

We computed the overdispersion of the place cell population or of individual place cells in each condition. We used the same technique as Fenton et al. (2010) to compute the population overdispersion applied on smoothed rate maps (not speed-filtered). We also computed the overdispersion of individual place cells similarly, only for cells that contributed at least 20 passes through the place field (to be able to evaluate the variance of the distribution of *Z* values relatively accurately). Paired statistics were performed comparing the average overdispersion per cell between conditions either for all cells or for cells with a place field on the goal that changes value. Overdispersion was calculated as follows (Fenton et al., 2010): the entire session was divided into 5 s episodes and then an expected number of spikes was computed per episode, using the following formula:

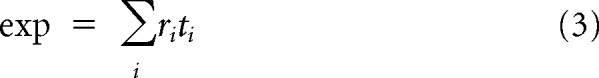
 where *r_i_* is the firing rate at location *i* and *t_i_* is the time spent in location *i* during this pass. Only passes with at least 5 expected action potentials (*exp* ≥5, equivalent to 1 Hz) were considered for the analysis because these would reflect passes through the place field. For each pass, the normalized SD of the expected number of spikes, *Z*, was computed as follows:

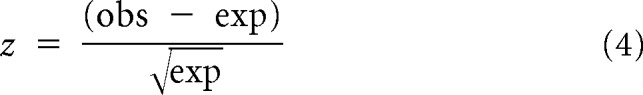
 with obs representing the observed number of spikes during the pass. Finally, the overdispersion value was computed as the variance of the distribution of *Z* values for all passes. As mentioned above, overdispersion was computed either for the whole place cell population (all passes combined together for a given session type, each cell contributing once per session type using the instance with the higher mean firing rate) or per cell (similarly, only one instance of each cell per session type was used).

##### Analyses of goal activity.

As with the behavior-related analysis, we first compared the firing rate at the goal zones (defined spatially) to the firing rate at control goal zones, for low or high speeds. We also calculated a spatial firing preference index, defined temporally, to evaluate a spatial bias of goal firing as follows:

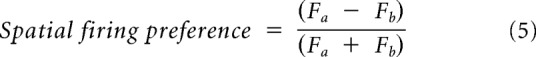
 where *F_a_* is the firing rate during left goal visits and *F_b_* is the firing rate during right goal visits. To address the question of value coding at the population level, we first compared the firing rate at the high value goal zone with that of the low value goal zone. We also calculated a value firing preference index for the 1vs0 and 1vs3 sessions to evaluate goal firing as a function of the expected reward magnitude. This was calculated in the same way as the spatial firing preference index, where *F_a_* is the firing rate during trials at the high value goal zone and *F_b_* is the firing rate during trials at the low value goal zone.

##### Value coding.

To assess value coding at the single-cell level, we computed a similar value firing preference index, but between sessions and for each goal. *Fa* was the firing at the goal when it was modified (value = 0 or 3) and *Fb* was the firing at the same goal in the previous session (value = 1). This measured the relative amount of change in firing following the change in goal value. To determine whether this change was significant, we computed a shuffle distribution of value firing preference indices for the same cell and goal, in which the trials of the two conditions were shuffled. Only sessions with at least five visits to the goal were used and 5000 shuffle values were computed. The data were then compared with the shuffle distribution and deemed significantly higher than chance if they were higher than the 97.5th percentile or lower than chance if they were lower than the 2.5th percentile of the distribution (two-sided test). A given cell was termed transiently value-modulated if, at least once, it significantly increased or decreased its firing in the value session for at least one goal. To assess actual value-encoding of cells and not simple modulation by value, we considered whether cells were consistently encoding value: a cell was termed consistently value coding if it significantly changed its firing when value changed for the majority of sessions of the same type (and at least 2) and in the same direction (i.e., either increase or decrease of firing).

##### Peri-event time histograms (PETHs).

To assess whether activity at the goal had a specific temporal profile during the delay period, PETHs time-locked to goal activation were computed. Spike times for all trials in a session—that is, 2 s visits to the goal zone—were aligned with the feeder activation event flag and accumulated in 100 ms bins to produce a PETH covering 4 s before feeder activation to 2 s after activation. Then, each PETH was normalized by its maximum so that we could average PETHs from different cells. When the PETHs for different goals were combined (e.g., left and right), trials belonging to one or the other goal were combined for each cell and then averaged to form the PETH of that cell. When comparing the normalized firing rate during the delay, we used a bin of 1 s instead of 0.1 s. For the time–order analysis, the individual normalized PETHs from each cell were smoothed with a Gaussian filter of σ = 150 ms.

#### LFPs

Before analysis, direct current offsets, slowly changing components and running line noise were removed from the LFP data using the Chronux toolbox (Bokil et al., 2010) *locdetrend* function, which subtracts the linear regression line fit within a 1 s moving window, and the *rmlinesc* function, which removes significant sine waves based on their F-statistic. Data were also notch-filtered using a second-order digital filter at frequencies of 50, 150, and 250 Hz (MATLAB function *iirnotch*, Q-factor 100) and then resampled at 750 Hz using a polyphase anti-aliasing filter (MATLAB function *resample*, pchip interpolation).

Time–frequency spectrograms were generated using the MATLAB function *spectrogram* and were composed of 200 ms time windows with a 50% overlap. Analyses focused on the theta-frequency band (4–12 Hz). Running power for each frequency band was calculated as the mean power in that band, and frequency was calculated as the frequency associated with the maximum power. We then extracted these values during each goal activation event (4 s before, 2 s after) and aligned these windows to the event time point. These event-related spectral perturbations (ERSPs) were then averaged within each session and normalized by their mean and SD (i.e., *Z*-scored) with respect to random baseline events (Ahmed and Mehta, 2012; Donnelly et al., 2014; Nishida et al., 2014) so that consistent changes in spectral power or frequency across sessions could be assessed. We performed the same tests on data composed of session-averaged ERSPs and rat-averaged ERSPs for all sessions.

We similarly calculated the power spectral density estimate (PSD) of the LFP data, using the MATLAB *periodogram* function. This was computed for 500 logarithmically spaced points between 0 and 300 Hz using a Hamming window after data were zero-padded to the next highest power of 2. This method was applied to segments of the LFP truncated to include only the 4 s before and 2 s after each goal event. For each of these PSDs, we calculated the maximum power exhibited in the theta band and the frequencies associated with these maxima. We performed the same tests on data composed of session-averaged PSDs and rat-averaged PSDs for entire sessions (only session-averaged data are shown).

Statistical tests were conducted on the mean value per session within the time window; that is, low versus high (1vs0) theta power is the mean *Z*-scored theta power (*n* sessions long) in the low-value goal zone compared with the mean *Z*-scored theta power in the high-value goal zone (also *n* sessions long).

### Experimental design and statistical analysis

Unless stated otherwise, when all the data for a test were normally distributed (tested via *scipy.stats.normaltest* that uses skew and kurtosis), we ran parametric tests such as *t* tests, whereas nonparametric tests (e.g., Mann–Whitney test for nonpaired data, Wilcoxon signed-rank test for paired data) were used otherwise. The Wilcoxon signed-ranked test was also used to assess whether distributions of preference indices significantly differed from a distribution around a 0 mean. This test assesses whether the distribution of values is symmetrically distributed around 0. Boxplots were generally used to show data distribution, with the median as a black horizontal line, the first (Q1) and third (Q3) quartiles as the box limits, Q1 − 1.5 × interquartile range (IQR, Q3 − Q1) as the bottom whisker and Q3 + 1.5 × (IQR) as the top whisker. Distributions such as PETHs were compared with each other using the Kolmogorov–Smirnov test. To assess the level of correlation between two samples, we computed Pearson's *R* correlation coefficient (e.g., to test for remapping or for temporal order of cell firing). To compare proportions, we performed binomial tests.

### Histology

At the end of the study, rats were given a lethal dose of pentobarbital (Dolethal; Vetoquinol; 100 mg/kg, 1 ml, i.p.). The final position of the electrodes was marked by passing anodal current through one of the wires of each tetrode (15 μA for 30 s). Under deep anesthesia, the rats were perfused transcardially, first with a saline solution (NaCl 0.9%), then with a formalin solution (4%). Their brains were extracted and left in a 30% glucose solution for 1 or 2 d. Then, they were frozen with dry ice (carbon dioxide) and stored at −80°C. The frozen brains were cut at 30 μm intervals and stained with cresyl violet. They were examined under a light microscope to determine the cannula track and the final position of the electrodes. This information was combined with the distribution of neurons recorded per electrode depth to determine the putative limits of CA1 and CA3 hippocampal fields. Each unit was then associated to a putative hippocampal field (Fig. 3 shows the estimated trajectory of tetrodes for each rat).

## Results

In the description that follows we use the notation 1:0, 0:1 or 1:3, 3:1 to differentiate goal value on left versus right, respectively, and 1vs0 or 1vs3 if the specific goal location is not relevant.

### Behavior: rats' choices reflect goal value

Six implanted rats performed a total of 224 sequences of four sessions. Only the sequences providing exploitable neuronal signals were used for analysis (*n* = 117). Behavioral results are similar if all sessions, even those without exploitable units, are included (data not shown). The median number of 1vs0 sequences (1:0 or 0:1 sessions, interspersed with 1:1 sessions) per rat was 8.5 (max = 21, min = 5) and the median number of 1vs3 sequences (1:3 or 3:1 sessions, interspersed with 1:1 sessions) was 8 (max = 19, min = 4). Unless stated otherwise, the two 1:1 sessions were usually combined into 1vs1 and value-changing sessions 1:0 and 0:1 (1:3 or 3:1) into 1vs0 (or 1vs3, respectively). The 1:1 sessions in the middle of a sequence were not used because they could incorporate uncontrolled effects from the previous session.

An example of goal choice behavior from a 1vs0 sequence of four sessions is shown in Figure 1*D*, with the corresponding location of goal events (successful goal visits, at least 2 s spent in a goal location) and reward consumption. The goal visit rate, the number of goal events per minute, was 4.5 ± 0.15 (SEM across rats) in the 1vs1 condition, which is higher than previous studies (e.g., mean goal visit rate was 2.1 successful goal visits/min in Hok et al., 2007a). The average goal visit rate was 4.6 ± 0.1 visits/min in 1vs0 sessions and 3.7 ± 0.2 visits/min in 1vs3 sessions.

**Figure 2.**
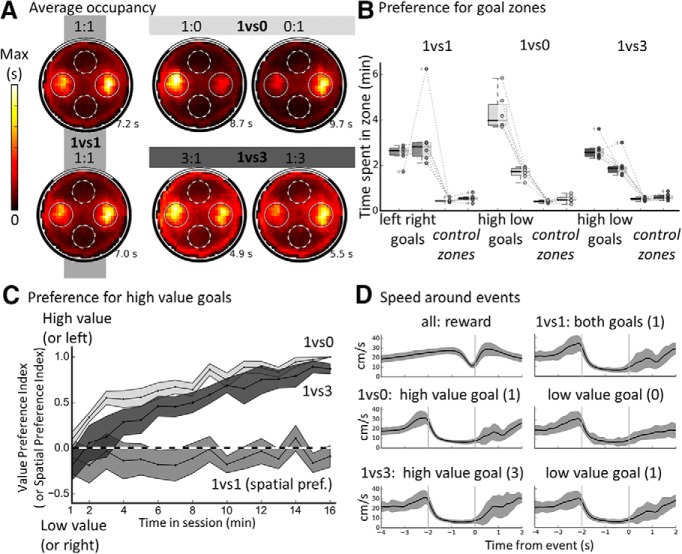
Behavior evidencing knowledge of the spatial, value, and temporal aspects of the task. ***A***, Occupancy maps averaged across all sessions of a given type per rat and then across all six rats. Goals are indicated as plain circles and control zones are indicated with dotted circles; max bin value is indicated in seconds for each map. Note the increased time spent at the goals, specifically higher value ones. A slight preference for the right goal is visible in 1vs1 sessions, but this is only due to one rat and not significant. ***B***, Average time spent per session type in goal areas and control goal areas. Value-changing sessions of a given type (1:0 and 0:1, 1:3 and 3:1) were combined (into 1vs0 or 1vs3, respectively). The data from each are shown as individual dots and a boxplot is shown per condition (see Materials and Methods). Note the strong bias toward visiting the goal zones compared with control zones and the bias toward goals of higher value when appropriate. ***C***, Within-session development of the value preference index computed over 1 min bins and averaged over sessions and then rats; preference for high value goal is shown for 1vs0 and 1vs3; spatial preference index is shown instead for 1vs1. Each point represents the average value for this bin and the shaded areas represent the SEM across the six rats. The dashed line indicates no preference. Note the absence of clear side preference in equal value (1vs1) sessions and the rapid emergence of a preference for high value in value-changing (1vs0 and 1vs3) sessions. ***D***, Instantaneous speed profile around recorded reward consumption (top left) or goal activation events. For the reward consumption event, 0 is the time when the rat was seen eating a food pellet. Note the clear (but brief) speed decrease around this time. For goal events, the goal period is shown surrounded by two vertical lines (from −2 to 0 s, 0 is the time of pellet dispenser activation). Note the increase of speed before arrival at the goal, the sharp decrease upon entry of the goal zone, the period of low speed at the goal, and finally the acceleration starting around the end of the delay period of 2 s. Average speed during the goal delay did not differ between conditions. For all speed profiles, SD across sessions is shown in gray and the scale is the same for all plots.

We then analyzed behavior both spatially and temporally. Spatially, we evaluated whether goal locations were more visited than two control nongoal zones. To visualize this, we plotted the cumulated occupancy maps for each of the conditions (Fig. 2*A*) averaged over sessions and then rats. All maps clearly show a high occupancy of the two goal locations in all conditions (including for the nonrewarded goal in 1vs0 sessions) and a preference for the goal of high value in value-changing sessions. Next, we compared the occupancy at the two goals (combined) to occupancy at the control zones (combined) for each condition (Fig. 2*B*): occupancy at the goals was always significantly higher than at the nongoal zones (1vs1: Wilcoxon, W = 0, *p* = 0.02; 1vs0: Wilcoxon, W = −11.4, *p* = 9.1 × 10^−5^; 1vs3: Wilcoxon, W = −10.7, *p* = 0.0001). In the 1vs1 condition, the occupancy for left and right goal was not different (Wilcoxon, W = 0, *p* = 0.75), showing an absence of spatial bias, whereas comparing the high-value goal with the low-value goal in the 1vs0 condition yielded a significant difference (W = 0, *p* = 0.027). This was also the case in the 1vs3 condition with a higher occupancy for the high-value goal compared with the low-value goal (Wilcoxon, W = 1.0, *p* = 0.046). This preference developed steadily across the course of a trial as shown by the behavioral value preference index computed per 1 min bins (Fig. 2*C*). The preference index should be ∼0 if there is no preference and toward 1 for a preference for the high-value goal. For 1vs1, the side preference is shown instead and a value of 1 would indicate a preference for the left goal. The time course of preference for the 1vs0 and 1vs3 conditions was compared with that of the side preference in the 1vs1 and was found to be significantly different (1vs0 vs 1vs1, Kolmogorov–Smirnov D = 0.94, *p* = 3.1 × 10^−7^; 1vs3 vs 1vs1, Kolmogorov–Smirnov D = 0.88, *p* = 2.32 × 10^−6^), whereas there was no significant difference between the profile of value preference for 1vs0 and 1vs3 conditions (Kolmogorov–Smirnov D = 0.38, *p* = 0.16). Therefore, rats' goal choice behavior was controlled by the number of pellets obtained and not by the spatial location of the goal zones. Overall, rats demonstrated learning of the spatial and value aspects of the task.

Temporally, we generated running speed profiles centered on the goal activation or the reward consumption event (Fig. 2*D*). For reward consumption events, the average speed and its SD appear to drop just before the event, indicating a relatively precise timing of the event. For goal events, velocity peaked just before goal zone entry (i.e., ∼2 s before pellet release) and then dropped steeply, indicating that the rats knew they were in the right zone. The average velocity during the goal period was not different for left or right goals in 1vs1 condition (left goal: 10.4 ± 1 cm/s, right goal: 10.2 ± 1.6 cm/s, *t*_(5)_ = 0.57, *p* = 0.59, paired *t* test) or high-value versus low-value goals (1vs0 condition, low-value: 10.1 ± 1.6 cm/s, high-value: 9.9 ± 1.4 cm/s, *t*_(5)_ = 0.27, *p* = 0.79; 1vs3 condition, low-value: 9.6 ± 1 cm/s, high-value: 9.8 ± 0.8 cm/s, *t*_(5)_ = −1.1, *p* = 0.33). Interestingly, even for trials in the 1vs0 condition when no pellet was released and no sound was emitted by the dispenser activation, the rats' speed increased again at the end of 2 s (Fig. 2*D*, middle, right inset), indicating processing of time as well as space (similar results were reported in Hok et al., 2007b, where the unique goal did not provide reward for 4 min).

To summarize, goal location, goal value, and temporal duration all influenced the animals' behavior in this task. Rats waited at the unmarked goal zones, indicating successful goal location processing. They also showed a strong preference for the higher value goal, indicating goal value processing, and they increased their running speed to exit the goal zone even when the goal was unrewarded, indicating an uncued awareness of temporal duration. Therefore, rats clearly processed the spatiotemporal and value components of the task.

### Electrophysiology: single units

We next looked at the activity of single pyramidal units to see whether there was evidence of goal encoding of the types seen previously and of goal value processing. Previous reports have observed a form of goal coding either from the place fields of CA1 place cells (Dupret et al., 2010) or from their out-of-field activity (Hok et al., 2007a). To investigate this, we first investigated whether place field or out-of-field activity encoded goal locations in 1vs1 sessions. Next, we analyzed this activity for putative value encoding using the value-changing sessions 1vs0 and 1vs3. Because of the well-established relationship of place cell activity to locomotor behavior (McNaughton et al., 1983), we analyzed activity both when the rat was actively engaged in goal processing (paused waiting at the goal, running toward the goal, possibly planning a trajectory, etc.) versus incidentally traversing the region during the reward–search phase.

**Figure 3.**
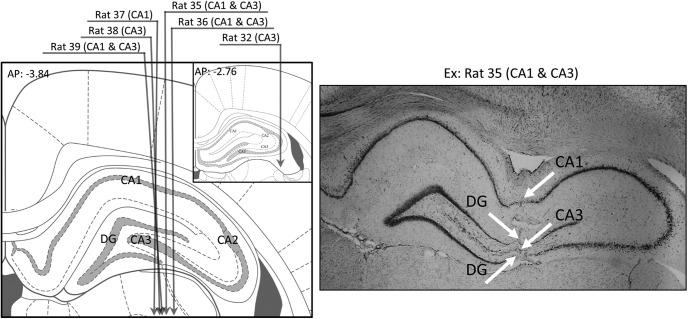
Histology. Left, Illustration of the estimated trajectory of the tetrode bundle for each rat. The verified coordinates of rat 32 were more anterior than planned (shown in inset). Right, Example histology slice for the rat that provided the most cells in CA1 and position of the layers crossed by the tetrode track.

**Figure 4.**
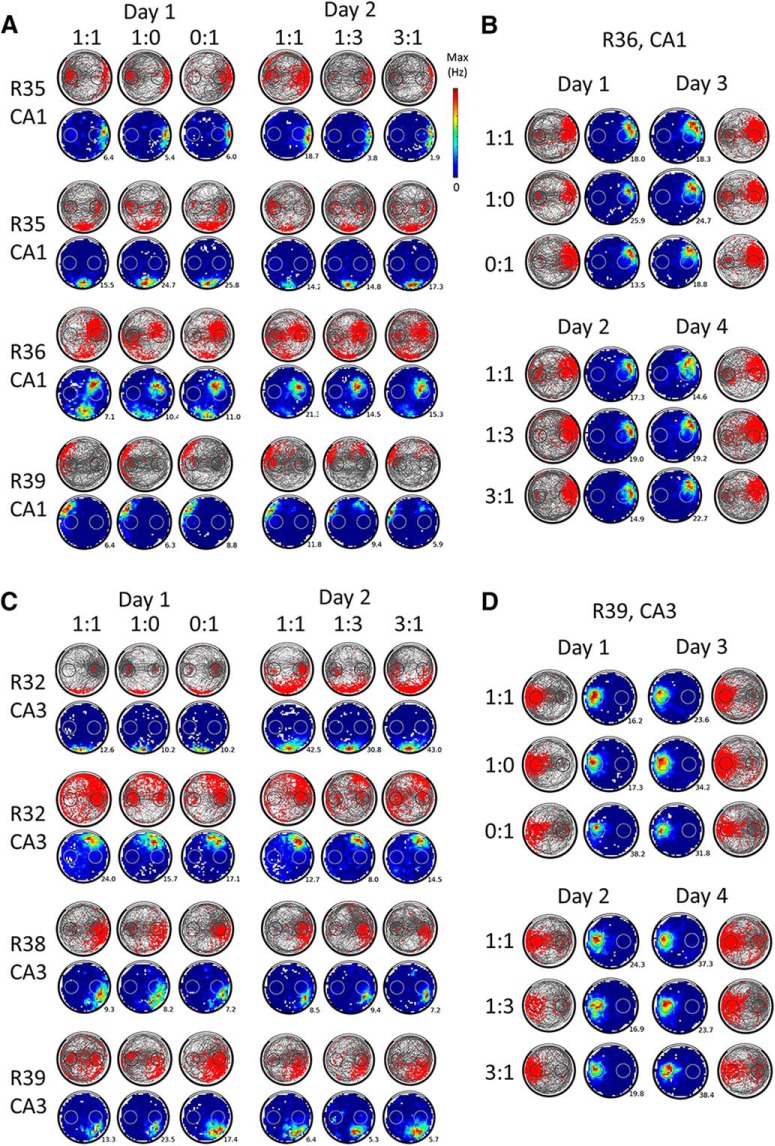
Place cells' activity remains spatially stable when goal value changes. ***A***, Activity of 4 example place cells from CA1 that were recorded for at least 2 d in the 1vs0 sequence and the 1vs3 sequence. The third session of each daily sequence (1:1) is not shown because it was not included in the analyses. Note that the order of the different sessions (or days) shown does not necessarily reflect the actual temporal order of the experiment (the position of the value-changing goal was counterbalanced between successive sessions of the same type as was the type of sequence). For a given cell, the spike plots (red spikes on gray trajectory) of each 16 min session are shown on the first row and the corresponding rate maps (average firing rate per bin, smoothed, with maximum firing rate indicated) are shown on the second row. Note how the rat behavior (visible on the spike plots) usually reflects goal values but how spatially stable the firing of the place cells remains within or across days regardless of changes in goal value. This is the case even for place fields located on a goal (e.g., third cell from the top). Also note the often increased spiking at the goal zones (outside of place fields), which contributes to the population goal-related signal. ***B***, Example of a CA1 place cell recorded during 4 different days, allowing for sampling of each value condition twice per goal. Spike and rate maps are shown as in ***A***. Note the stability of the spatial firing across different value conditions. ***C***, Activity of 4 example place cells recorded during at least 2 d from CA3, presented as in ***A***. ***D***, Example of a CA3 place cell recorded for 4 different days, presented as in ***B***. For CA3 cells, changes in goal value also do not appear to affect the firing of place cells.

A total of 157 unique putative pyramidal cells were recorded from the dorsal hippocampus of 6 rats performing the two-goal navigation task. Of these, 104 (66%, median = 10/rat, min = 3, max = 41) were categorized as place cells, of which 59 cells were considered to be from the CA1 hippocampal field (from 4 rats) and 45 cells from CA3 (from 5 rats). A summary of the histology results is shown in Figure 3 and example CA1 and CA3 place cells are shown in Figure 4.

The number of unique place cells that could be matched for several days were as follows: 2 d: 61, 3 d: 33, 4 d: 23, 5 d: 8, 6 d: 7, 7 d: 3 (see the Materials and Methods for the Tolias distance cross-day matching technique, relying on waveform similarities, and Fig. 4, *B* and *D*, for examples of cells recorded across 4 d). We also recorded 40 putative “silent cells” (Thompson and Best, 1989) that had scarce firing and no detected place fields (24% of pyramidal cells, median = 4/rat, min = 1, max = 22; 25 from CA1, 15 from CA3). Because their firing was very low by definition (median speed-filtered firing = 0.06 Hz), we did not include them in the present analysis, but report that most of their firing was concentrated at the goal locations.

### General CA1 and CA3 differences

First, we compared the general properties of CA1 and CA3 place cells (Fig. 4*A*,*C* for examples of each). CA1 cells had a shorter waveform width (median = 500 μs) compared with CA3 cells (median = 531 μs, Mann–Whitney *U* = 920, *p* = 0.0068) and the average firing rate of CA1 place cells (median = 0.5 Hz) was lower than that of CA3 place cells (median = 1.1 Hz, Mann–Whitney *U* = 815, *p* = 0.0008). The 104 recorded place cells had 134 place fields and the mean number of place fields per cell was not significantly different between CA1 and CA3 (1.4 for CA1 cells, 1.7 for CA3 cells, Mann–Whitney *U* = 1920, *p* = 0.12). However, CA3 place fields had a higher mean firing rate and peak firing rate compared with CA1 fields (median firing of 73 CA1 place fields = 4.1 Hz, median firing of 61 CA3 place fields = 5.7 Hz, Mann–Whitney *U* = 1781, *p* = 0.046; median of all CA1 peak place field firing = 9.2 Hz, median of all CA3 peak place field firing = 12.6 Hz, Mann–Whitney *U* = 1726, *p* = 0.025). CA3 fields were also generally larger than CA1 ones (median size of CA1 place fields = 35 pixels, median size of all CA3 place fields = 75 pixels, Mann–Whitney *U* = 1505.5, *p* = 0.0013).

### Place fields do not overrepresent goal locations

**Figure 5.**
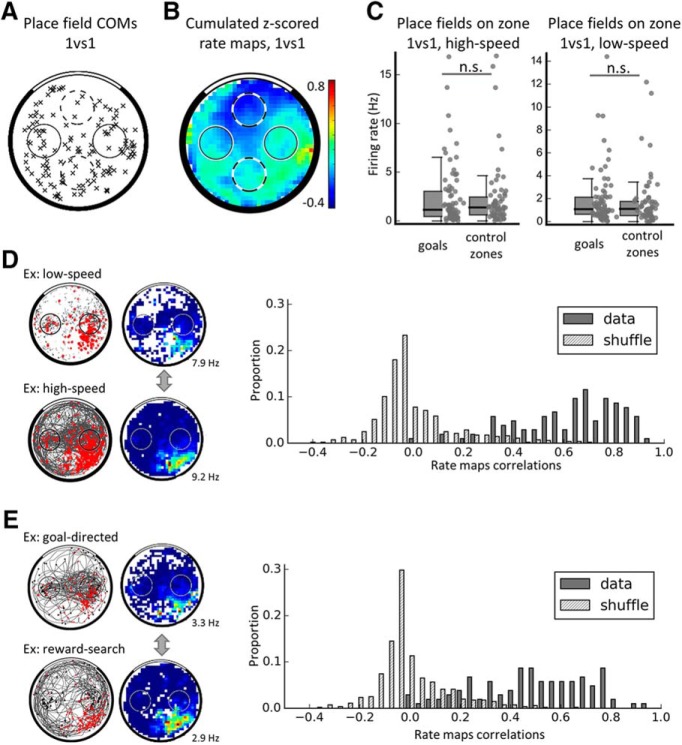
Place fields do not overrepresent the goal locations in the equal value (1vs1) condition. ***A***, Distribution of the COMs of all place fields in the 1vs1 condition. Place fields did not significantly overrepresent goal locations (black circles) compared with control zones (dashed circles). ***B***, For illustration purposes, cumulative firing map in the 1vs1 condition (average *Z*-score in each location) using the *Z*-scored speed-filtered map of each place cell. Note that firing rate does not seem to increase specifically at the goals. Average *Z*-score is indicated. ***C***, Firing rate of place fields compared between goals and control nongoal zones either for high speeds (>15 cm/s, left) or low speeds (<15 cm/s, right). Individual data points are shown as well as boxplots (which are as in Fig. 2*B*). Place fields do not increase (nor decrease) their firing at the goals compared with the nongoals. ***D***, Correlations of all (smoothed) place cell maps at low versus high speed compared with a shuffle distribution. The data are more correlated than chance and centered on high correlation values; therefore, there is no global remapping between low speeds and high speeds. An example cell from CA3 is shown on the left. ***E***, Similar correlations as ***D*** but between goal-directed versus reward-search maps, with reward events shown as stars and goal events shown as triangles. The example is from the same cell as in ***D***. Again, the distribution of correlations from our dataset are significantly higher than chance; therefore, there is no global remapping of place cells between these two task phases.

To assess whether place cells would specifically represent the goal locations with their place fields, we first analyzed place-specific firing when the rats were moving across the arena (speed >15 cm/s), focusing on the 1vs1 condition. The 134 place fields were distributed across the entire arena (Fig. 5*A* shows the COMs of all place fields). We analyzed either all place fields or only the largest place field per cell (*n* = 101). First, we computed a goal representation percentage using the number of COMs in the goals and those located in equivalent control zones (see Materials and Methods). Place fields did not overrepresent the goal zones (when only place fields in the goal or control zones were considered, 64.7% of 34 COMs were closer to the goals, n.s. compared with shuffled distribution; when all place fields in the arena were considered, 56% of 134 COMs were closer to the goals, n.s. compared with shuffle distribution). A similar absence of goal overrepresentation was found if only the biggest place field for each place cell was taken into account (goal representation percentage of 67% of 27 place fields in a goal or control zone, n.s. compared with shuffled distribution; goal representation percentage of 56% out of 104 place fields in the arena, n.s. compared with shuffle distribution) or if the CA1 and CA3 populations were tested separately. Similarly, comparing the number of cells that have a place field encroaching on either of the goals (*n* = 78) with that of cells with a place field on any of the control nongoal zones (*n* = 66) did not yield a significant difference (binomial test, *p* = 0.35). We also used an analysis adapted from Dupret et al. (2010), which did not yield any overrepresentation of goals either (data not shown). Finally, we noticed that CA1 place fields seemed more peripheral than CA3 ones and compared the distance to the center of the arena of CA1 and CA3 COMs. We found that CA3 fields were indeed more central, either when all place fields were used (mean distance to arena center: 27.2 ± 7.2 cm for 73 CA1 place fields, 22.5 ± 8 cm for 61 CA3 place fields, Mann–Whitney *U* = 1393.0, *p* = 0.0002) or when only the biggest place fields (1 per place cell) were used (mean distance to arena center: 29.2 ± 7 cm for 59 CA1 place fields, 23.2 ± 7.8 cm for 45 CA3 place fields, Mann–Whitney *U* = 783.0, *p* = .0004). Because this difference was probably due to the difference in place field size between the two populations (larger place fields should have a more central COM), we did not analyze it further.

To conclude, place fields did not overrepresent the spatial goals in this task, which is consistent with previous findings in the single goal version of the task (Hok et al., 2007a) and in contrast to other findings (Hollup et al., 2001; Dupret et al., 2010; H Lee et al., 2012) in which the overrepresented location was also a directly rewarded location.

Place cells might signal goal locations not through the presence or absence of a place field, but rather via modulations of firing rate. Therefore, we compared the firing rate between goal and nongoal zones, when place fields were found to overlap on the zone of interest and when the rat was running (speed >15 cm/s). For illustration purposes, we averaged the speed-filtered rate maps of all recorded place cells (normalized by *Z*-scoring first) and no goal overrepresentation was visible (Fig. 5*B*). Because there was no difference between the mean firing at the two goals (Mann–Whitney *U* = 983.0, *p* = 0.23), the firing rates were combined, and similarly for the two nongoals (Mann–Whitney *U* = 649.0, *p* = 0.562). No difference in firing rate was observed between goals (median = 1.36 Hz, *n* = 78) and nongoals (median = 1.12 Hz, *n* = 66, Mann–Whitney *U* = 2432.5, *p* = 0.57). The same analysis was applied on low-speed data (e.g., when the rat was waiting at the goal zone or elsewhere) and also did not yield any difference in firing rate within place fields (median firing at goals: 1.15 Hz, nongoals: 1.35 Hz, Mann–Whitney *U* = 2492.0, *p* = 0.743; Fig. 5*C*).

Finally, we considered whether place fields might remap depending on task conditions; that is, when the rat was waiting (speed <15 cm/s) versus moving (speed >15 cm/s), or searching for food (reward-search phase) compared with approaching the goal followed by waiting at the goal (goal-directed phase, see Materials and Methods). For this, we computed Pearson's correlation coefficients for each place cell between its rate map at low speeds versus high speeds (cf. Fig. 5*D*) or its rate map during foraging versus goal-directed phases (Fig. 5*E*). A shuffled distribution of correlations was computed in each case to estimate the distribution of a randomly remapping population by computing correlations between different cells recorded during the same session (see Materials and Methods). In both cases, the shuffled distributions were different from the data, the latter showing higher correlations (for the speed-related correlations: Kolmogorov–Smirnov D = 0.88, *p* = 9.5 × 10^−62^, median of shuffle = −0.04, 95^th^ percentile of shuffle distribution = 0.32, median of data = 0.67; for the foraging vs goal-directed correlations: Kolmogorov–Smirnov D = 0.81, *p* = 1.6 × 10^−52^, median of shuffle = −0.04, 95^th^ percentile of shuffle distribution = 0.29, median of data = 0.5). We also tested whether the distribution of correlations were different from unimodality because a bimodal distribution of correlations could indicate that different subpopulations of place cells behaved differently – that is, partial remapping. For this, we used Hartigan's dip test (Hartigan and Hartigan, 1985) and found that none of the two distributions of correlations was significantly different from a unimodal distribution (correlations between different speed phases, dip statistic = 0.028, *p* = 0.817; correlations between different task phases, dip statistic = 0.028, *p* = 0.884). Finally, we individually looked at the maps with low (<0.2) correlation values to assess whether this was due to individual cells remapping. In ∼50% of the cases, the place field location was not sampled enough in one of the conditions, spuriously creating a low correlation. In ∼45% of the low correlation cases, the maps had relatively low firing and the correlation values could probably not be very accurately computed. In 9% of cases and only for goal-directed versus reward search comparisons, we found that the place field disappeared in one of the conditions, but this comprised only three cells (zero in the speed-based correlations) and we do not believe this to be of any significance. Therefore, the place cell population did not globally remap between the different behavioral phases. At first glance, this seems to contrast with several studies that previously found task-based remapping (Wiener et al., 1989; Markus et al., 1995; Siegel et al., 2008; Ainge et al., 2012); however, the task phases compared in these studies were always performed in blocks. In tasks that alternate two types of behavior, like the present study, no remapping is found (Trullier et al., 1999; Zinyuk et al., 2000; Siegel et al., 2008).

Overall, our results so far indicate that the two spatial goals are not overrepresented by place fields and that the population activity is encoding space in a homogeneous manner regardless of the moment-to-moment behavior or putative intention of the rat.

### Is there out-of-field spatial goal coding?

As established in previous studies (Hok et al., 2007a,b, 2013), place cells express goal-related activity in the form of increased out-of-field firing at the goal, in a single goal version of the continuous navigation task. This signal is sparse at the single-cell level but clear at the population level. Therefore, we investigated whether goal-related firing was present in our task by analyzing out-of-field activity at the goal, either in its firing rate or its temporal aspect. First, we analyzed the out-of-field firing rate of place cells at goal locations (i.e., when no detected place field, whether primary, secondary, or other, was encroaching on the goals) compared with similar out-of-field firing occurring at the control zones. At low speeds, the firing at the goal was significantly higher than in the corresponding nongoal zone (right goal, median = 0.22 Hz vs control zone, median = 0.12 Hz, Mann–Whitney *U* = 1266, *p* = 0.0012; left goal, median = 0.33 Hz vs control zone, median = 0.10 Hz, Mann–Whitney *U* = 1108, *p* = 0.0009). The two goal zones were not combined because their firing was different (Mann–Whitney *U* = 1179, *p* = 0.02); however, the firing of the two control zones was not different (Mann–Whitney *U* = 2005, *p* = 0.62). In the case of speeds >15 cm/s—that is, when the rat was just crossing the zones without stopping—there was no difference between the out-of-field firing at goals (median = 0.12 Hz) and nongoals (median = 0.10 Hz, Mann–Whitney *U* = 3569, *p* = 0.21; the firing at both goals or nongoals was not different and combined). Therefore, goal-related activity—that is, out-of-field firing at the goal—seems to be expressed only at low speeds, presumably during the waiting period. This result was reproduced when looking separately at CA1 and, importantly, CA3 place cells: goal-related activity was present in both cases (for 55 CA1 place cells, median goals = 0.3 Hz, median at control zones = 0.18 Hz, Mann–Whitney *U* = 1100, *p* = 0.014; for *n* = 38 CA3 place cells, median at goals = 0.29 Hz, median at control zones = 0.08 Hz, Mann–Whitney *U* = 377, *p* = 0.01). Therefore, we replicate the goal-related finding seen in the single goal version of the task (Hok et al., 2007a) and extend it to CA3 place cells. Finally, we tested whether goal-related activity, for place cells without a field on any of the goal zones (*n* = 26), had a bias toward a specific goal. To do so, we computed the side firing preference index in 1vs1 sessions: its median was 0.05 and the distribution was symmetric around 0 (Wilcoxon signed-rank test, T = 138.5, *p*= 0.35). Therefore, both goals were homogeneously represented.

### Temporal characteristics of activity during the delay period in the equal-value condition

**Figure 6.**
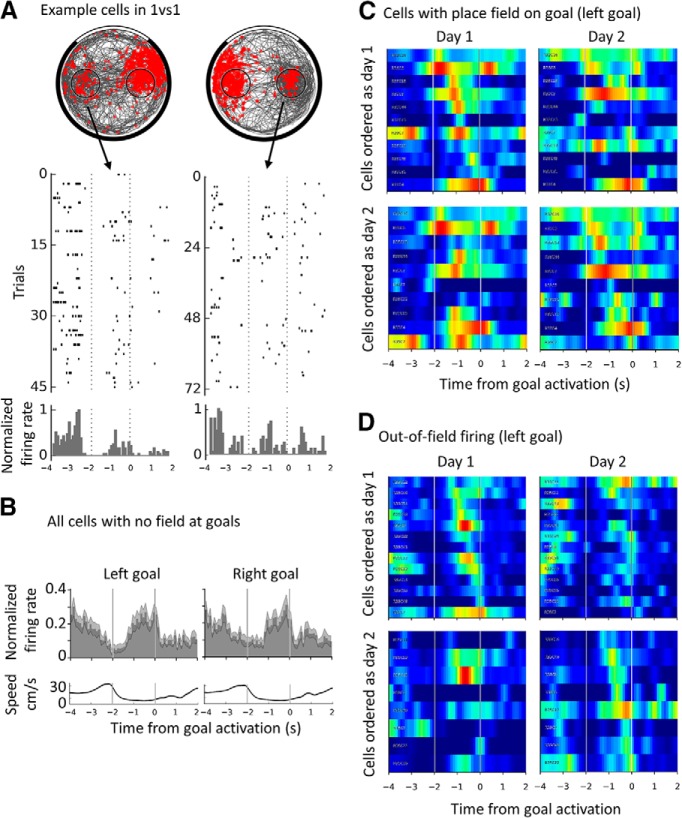
Increase in out-of-field firing rate across the goal delay period and absence of time coding. ***A***, Left, Example spike plot, raster plot and corresponding normalized PETHs for a CA1 cell recorded during a 1vs1 session, showing out-of-field activity at the left goal (and a place field on the right goal). The goal delay is shown by two vertical dashed lines. Right, Similar example but for a CA3 cell showing out-of-field activity on the right goal (and a place field on the left). ***B***, Temporal profile of goal-related activity (normalized firing during correct goal trials) for place cells with no field on any goal (*n* = 26) for the left and the right goal. The goal delay is indicated by the two vertical lines. Note the sharp decrease of firing upon entry in the goal zone and the increase during the delay period, followed by an abrupt drop just after the activation of the pellet dispenser. Shaded area indicates SEM. The corresponding average speed profile is shown below each plot. ***C***, Individual PETHs around the left goal for place cells with a field encroaching on that goal with a line per cell. Repeated recordings of the same cells are shown on the left and right; cells are ordered either as a function of the time of peak firing during the delay period during day 1 (top) or day 2 (bottom). Note that applying the same order to the same cells recorded during a different day (left vs right) strongly disrupts the apparent order of firing. Data for the right goal are not shown but are equivalent. The color scale is as for previously shown rate maps, with red indicating a maximum normalized firing of 1 and blue indicating no firing. ***D***, Similarly to ***C***, individual PETHs for out-of-field firing at the left goal. The same phenomenon is observed, namely, the apparent order of peak firing during the goal delay for a given day is not reproduced on a different recording day.

We then aimed to describe the temporal aspects of the goal activity and to evaluate possible time coding during the goal delay period. For this, we combined the CA1 and CA3 data and looked at the activity before (−4:−2 s), during (−2:0 s), and after (0:2 s) the delay at the goal, *t* = 0 being the pellet dispenser actuation. Only sessions with at least one visit to each goal were included in this analysis. Examples of raster plots and normalized PETHs from two place cells are shown in Figure 6*A*. Individual PETHs were then averaged over all place cells with no field on any of the goal zones (*n* = 26), shown in Figure 6*B* for each goal separately (top). As observed in previous one-goal versions of the task, the averaged PETHs at the goal showed a drop in firing upon entry in the goal and then an increase of firing ∼1 s after entry in the zone. The population activity at the goal tended to reach its peak after 1 s spent in the goal zone, consistent with previous results (Hok et al., 2007a). We compared the activity profile of the two goals and they were not significantly different (Kolmogorov–Smirnov D = 0.13, *p* = 0.62). Therefore, we combined the PETHs from the two goals and compared the normalized firing rate during the first and second halves of the delay. The firing rate significantly increased during the delay (median [−2:−1] = 0.23, median [−1:0] = 0.65, paired Wilcoxon test, W = 4, *p* = 1.3 × 10^−5^). Finally, we performed the same comparison when all place cells were taken together (*n* = 101) regardless of place field position. In this case, the left and right distributions were different (Kolmogorov–Smirnov D = 0.38, *p* = 0.0002), but the same increase during the delay was found, for the left goal (median [−2:−1] = 0.26, median [−1:0] = 0.57, paired Wilcoxon test, W = 710.5, *p* = 7.33 × 10^−10^) or the right goal (med [−2:−1] = 0.36, med [−1:0] = 0.57, paired Wilcoxon test, W = 1206, *p* = 1.55 × 10^−5^). Incidentally, we also looked at the temporal profile around the reward consumption event (which could take place anywhere in the arena): in contrast to firing during the goal period, no notable variations in the firing of place cells were observed, whether all firing or only out-of-field firing was considered (data not shown). Therefore, the activity of place cells is temporally organized during the goal delay, increasing as the rat waits at the goal.

We also looked for evidence of temporally organized firing peaks representing the whole of the 2 s waiting period, consistent with previous reports of time cells in the hippocampus (MacDonald et al., 2011). To this end, we smoothed the PETH bins for each cell (bins = 0.1 s, Gaussian filter of σ = 0.15 s) and computed the time of maximum firing for each cell and goal separately during the 2 s goal delay. Time coding would imply a reproducible activity at specific times during the delay whenever the animal was waiting at that goal. We tested this for out-of-field firing and for place field firing separately. For the out-of-field firing, we could compare the repeatability of the order of firing at the left versus the right goal. For place field firing, we compared it across days for several recordings of the same cell. First, we organized the individual PETHs of cells by their time of peak during the goal delay, including only the cells with normalized out-of-field firing PETHs that had a peak during the goal period (>0.3). This ranking revealed a fairly continuous representation of all times during the delay period (Fig. 6*D*, top left, for the out-of-field firing at the left goal during a daily 1vs1 session). However, imposing the cell order for one goal on the other goal (data not shown) disrupted the temporal order of the neurons, indicating that, if cells encode a specific time with their out-of-field firing, then it must be different for the two goals (possibly indicating retiming of the cells; MacDonald et al., 2011). To quantify this reordering, we computed the correlation between the order of cell numbers (from 1 to *n*) and the time of the firing peak (in bins from the start). The correlation for the left goal, setting the order of cells, was high as expected (*r*_(7)_ = 0.95, *p* = 8.1 × 10^−5^) but nonsignificant when the same order was applied to the other goal (*r*_(7)_ = −0.08, *p* = 0.8). Therefore, activity at the goal might seem to show peaks at all times during the delay, but the time of the peaks is not consistent between goals even though the delay at both goals is always the same. This may reflect spatial inconstancy during this waiting period because the animal could still move around within the goal zone.

To test this further and assess place fields as well, we investigated whether the temporal order of firing was conserved between days. We compared the order of goal firing peaks between two 1vs1 sessions from different days where the same cells were recorded (sessions might differ for each cell, but are termed day 1 and day 2 for simplicity). First, we analyzed the activity of cells with a field at the goal (see Fig. 6*C* for the left goal cells). For the left goal, the correlation between cell order and time of maximum firing was significant on day 1 but not day 2 (*n* = 11, *p* = 2.3 × 10^−7^ and *p* = 95, respectively); similar results were obtained for the right goal (*n* = 10, *p* = 6.5 × 10^−5^ and *p* = 0.53). This same pattern of results was observed for cells with no field on the goal in question (left goal out-of-field firing is shown in Fig. 6*D*, left, *n* = 13, *p* = 6.1 × 10^−11^ and *p* = 0.64, right, *n* = 11, *p* = 8.3 × 10^−8^ and *p* = 0.14). To summarize, the activity of place cells at the goal is temporally organized insofar as firing increases ∼1 s during the goal delay, but we did not see evidence of consistent, homogeneous time coding.

### Is there goal value coding?

Next, we focused on the conditions with different goal values to assess whether hippocampal cells encode the value of spatial goals in this task (see Fig. 4 for an example of the activity of several cells in the different value conditions). We decided to address this question with three approaches. First, at the population level, we investigated whether place fields would change their activity depending on goal values (by shifting their location, modulating their firing rate, or via changes of moment-to-moment firing). Second, we analyzed out-of-field goal firing to determine whether it would globally increase or decrease for a specific goal value. Third, at the single-cell level, we tested whether individual place cells with a field on the goal would consistently encode specific goal values (out-of-field firing is individually too sparse to be analyzed per cell).

First, to assess whether value-changing sessions would trigger remapping or field instability, we computed the correlation for each cell between each session and all the subsequent ones either for this day or the other days where this cell was recorded. The average correlation coefficient over all cells and sessions was 0.7 ± 0.085 (SD), so maps were overall very stable (min = 0.45, max = 0.80). The average correlation between the first equal-value session and all subsequent value-changing sessions was 0.69 ± 0.02 and the average correlation between the first equal-value session and all subsequent equal-value sessions was also 0.69 ± 0.02. Therefore, there was no difference in the stability of place cells between equal value or value-changing sessions. Overall, we did not detect any visible pattern linked to value-changing sessions (data not shown, but see examples in Fig. 4). Therefore, general place cell activity was very stable spatially across sessions regardless of changes in goal value.

**Figure 7.**
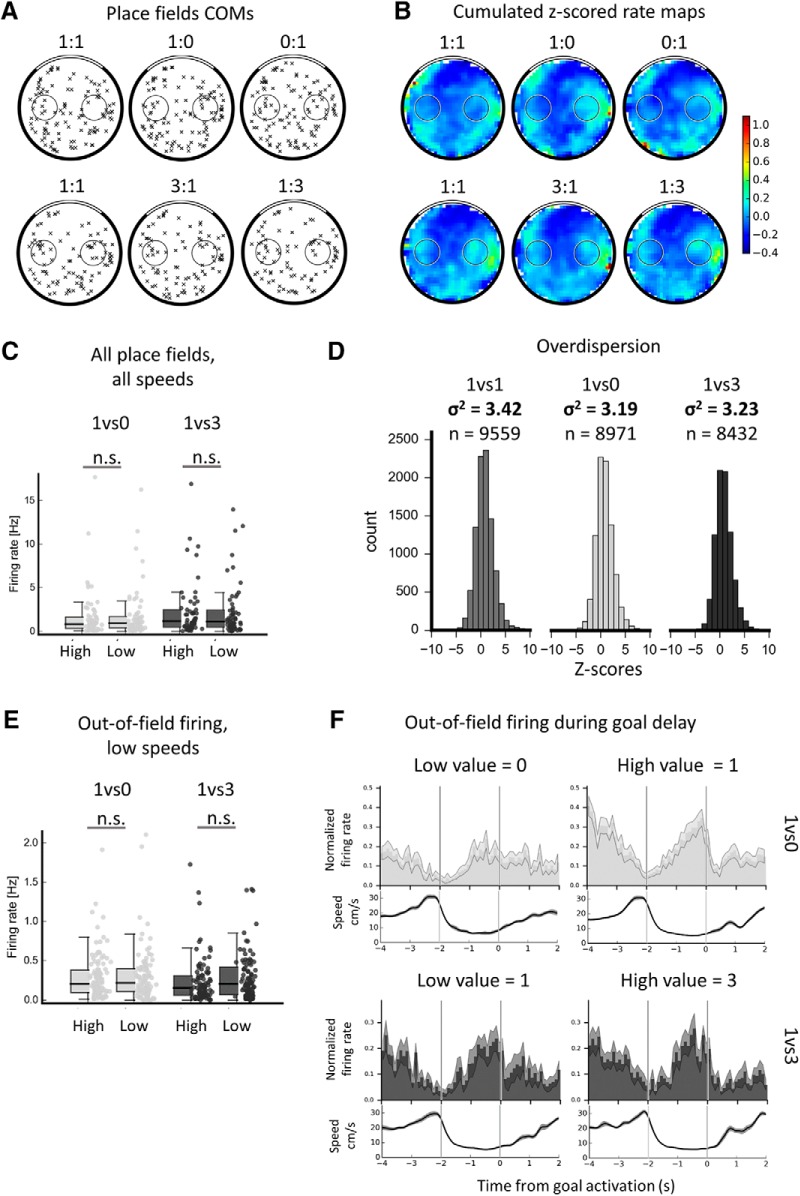
Insensitivity of the population of hippocampal cells to goal value. ***A***, Place fields' COM distribution in all conditions. Top, Distribution of all place fields recorded in a 1vs0 sequence (from *n* = 88 place cells). Bottom, Distribution of all place fields recorded in a 1vs3 sequence (from *n* = 74 place cells); place fields were not found to overrepresent the high-value goal. ***B***, For illustration, cumulated (averaged) *Z*-scored speed-filtered rate maps in all conditions (top, 1vs0 sequence, *n* = 88; bottom, 1vs3 sequence, *n* = 74); note how similar the pattern of firing appears across different value conditions. ***C***, Firing rate for all place cells with a field on a given goal zone grouped by goal value (high or low) in the 1vs0 or 1vs3 conditions for all speeds. No difference of firing was found between the high- and low-value goals. ***D***, For illustration, overdispersion of the population of place cells in each condition. The numbers of passes through a place field used to build the distribution are indicated for each condition; overdispersion values are very similar for all conditions. ***E***, Out-of-field firing rate of all place cells without a field on a given zone, as in ***D***, only for low (<15 cm/s) speeds. No difference was found between the two goals. ***F***, Normalized PETH (constructed as above) of place cells with no fields at either of the goal zones, for the low-value (left) or high-value (right) goals in the 1vs0 (top) or 1vs3 (bottom) condition. Note the reproducibility of the previously observed increase of firing during the goal period. The only difference found was between the high- and low-value profiles in the 1vs0 condition.

The place fields' COMs were computed for both types of value-changing sessions and their spatial distribution is shown in Figure 7*A*. Using the same shuffle analysis as previously (see Materials and Methods), we assessed whether COMs inside or closest to the high-value goal were more numerous than COMs inside or closest to any of the control zones or to the other, lower-value, goal. None of the value configurations exhibited any form of goal inhomogeneity (cf. Fig. 7*A*).

Next, we investigated whether place cells would encode goal value via modulations of their place field firing rate. We compared firing rates at the high-value goals with those at the low-value goals, including only data when a place field was encroaching on a given zone. This is illustrated by the cumulated rate maps for all recorded place cells shown in Figure 7*B*. For both value configurations, cells fired similarly in the low- and high-value goal zones (1vs0, low-goal vs high-goal Mann–Whitney *U* = 2092, *p* = 0.70; 1vs3, low-goal vs high-goal Mann–Whitney *U* = 1613, *p* = 0.90; Fig. 7*C*). The same comparison yielded similar results when restricted to low- or high-speed data (data not shown).

Last, we sought a way to analyze the trial-to-trial variability of place cells' firing in the different value conditions to assess whether, even though place fields appeared very stable, there could be more subtle variations in firing between equal value and value-changing conditions. The overdispersion measure, which is the variance of the distribution of *Z*-values for each pass through the place field, allowed us to quantify this. First, to compare our results with the existing literature, we computed the overdispersion of the population of place cells recorded in each condition using a method described previously (Fenton et al., 2010). We found that the overdispersion in the 1vs1 condition was 3.42 (Fig. 7*D*, left), which is very similar to values observed in comparable tasks in the literature (Fenton et al., 2010; Hok et al., 2013). Interestingly, we also obtained very similar values in the value-changing conditions (Fig. 7*D*, middle and right): the overdispersion was 3.19 for 1vs0 and 3.23 for 1vs3. To assess whether there could be an effect of goal value changes on the overdispersion of the firing of individual place cells, we performed the overdispersion computation on individual cells. To obtain a sufficiently accurate estimate of overdispersion, we only used place cells that provided at least 20 passes. We first compared the overdispersion of CA1 and CA3 place cell firing and found no difference between these two values in any of the conditions (1vs1, 1vs0, or 1vs3; data not shown); therefore, CA1 and CA3 place cells were combined for the rest of the analysis. We then used a paired design comparing the overdispersion for value-changing sessions to that of the corresponding (i.e., for the same cell and day) equal-value session. In the 1vs0 condition, there was no difference of overdispersion compared with the corresponding 1vs1 sessions (med 1vs1 = 2.6, med 1vs0 = 2.4, paired Wilcoxon signed-rank test, T = 391, *p* = 0.10 for *n* = 46 place cells). Similarly, no difference was found between 1vs3 sessions and the corresponding 1vs1 sessions (med 1vs1 = 2.4, med 1vs3 = 2.6, paired Wilcoxon signed-rank test, T = 448, *p* = 0.15 for *n* = 48 place cells). This analysis included all place cells, whereas perhaps only the cells with a place field close to the goal with a changing value would be affected by the change. We repeated the analysis using only cells with a place field on the changing-value goal (*n* = 24 cells for 1vs0, *n* = 27 cells for 1vs3). The same results were observed: there was no significant effect of changing the goal value on the overdispersion of place cell firing (med 1vs1 = 2.8, med 1vs0 = 2.9, paired Wilcoxon signed-rank test, T = 140, *p* = 0.78 for *n* = 24 place cells; med 1vs1 = 2.7, med 1vs3 = 2.9, paired Wilcoxon signed-rank test, T = 179, *p* = 0.81 for *n* = 27 place cells). In conclusion, the overdispersion of place cells does not significantly change between different value conditions. This is possibly in contrast with a report from Wikenheiser and Redish (2011), in which changes in reward contingencies modulated the overdispersion; however, in their task, the change in reward contingency probably modified the strategy to use (i.e., always skip a goal instead of stopping at each goal) and the differences in overdispersion could be due to this change of strategy, possibly related to changes in spatial attention.

Next, we addressed the question of value coding by the out-of-field firing of place cells. To do so, we combined CA1 and CA3 data and first focused on the firing at the two goals of different value in all the speed conditions (see Fig. 7*E* for low speeds). Even though significant goal firing had been found in the 1vs1 condition for low speeds, there was no difference between the firing rate at high-value goals versus low-value goals in the 1vs0 condition (*n* = 104 cells, Mann–Whitney *U* = 5249, *p* = 0.70) nor in the 1vs3 condition (*n* = 92 cells, Mann–Whitney *U* = 3727, *p* = 0.20). As another approach to assessing a possible value coding of goal-related activity, we computed the value firing preference index (see Materials and Methods). This only included place cells with no place field at any of the goals. The median value firing preference index was <0.001 in 1vs0 conditions (*n* = 24 place cells) and was not different from 0 (Wilcoxon signed-rank test, T = 120, *p* = 0.39), indicating that cells were not firing more at any of the goals (in particular, the firing did not increase for the high-value goals). Similarly, the median index was −0.06 in the 1vs3 condition (*n* = 19 place cells), which was not different from 0 (Wilcoxon signed-rank test, T = 74, *p* = 0.39). Therefore, the goal firing was not biased toward a specific goal value. Overall, neither place fields nor out-of-field activity encoded goal value at the population level, indicating that goal-related activity in particular is unlikely to be reflecting reward expectation.

#### PETH data

We next investigated whether the temporal profile of out-of-field goal-firing would be different depending on goal value. Focusing first on cells with no place field at either of the goals, low vs high value PETHs for the 1vs0 condition (*n* = 24) yielded a significant difference over the whole PETH period (Kolmogorov–Smirnov D = 0.33, *p* = 0.0018), but no difference was observed in the 1vs3 condition (*n* = 19, Kolmogorov–Smirnov D = 0.09, *p* = 0.90). Focusing on the 1vs0 condition, in both goal zones, neurons increased their firing rate during the second half of the delay period (*t*_(23)_ = −7.9, *p* = 4.8 × 10^−8^, *t*_(23)_ = −8.1, *p* = 3.8 × 10^−8^, respective paired *t* tests), reproducing the results for the 1vs1 condition. Also for the 1vs0 condition, in the one-pellet goal zone, neurons decreased their firing rate as the rat exited the zone (normalized firing rate for [−1:0 s] = 0.68 ± 0.3, for [0:1 s] = 0.32 ± 0.2, *t*_(23)_ = 5.6, *p* = 9.6 × 10^−6^, paired *t* test). In the goal zone yielding no pellets, this same decrease was present, but of visibly smaller amplitude (see Fig. 7*F*, top left; normalized firing rate for [−1:0 s] = 0.6 ± 0.3, for [0:1 s] = 0.47 ± 0.3, *t*_(23)_ = 67, *p* = 0.03, paired *t* test). A possible explanation for this could be the absence of the pellet dispenser sound at the end of the unrewarded delay period. This is only of interest if one focuses on the activity outside of the delay period, which we did not aim to do, but could indicate sustained goal firing. Overall, the analysis of goal-related activity PETHs from the value-changing conditions confirms the increase in overall firing during the delay period regardless of goal value and possibly suggests some influence of the absence of expected reward on the temporal profile of goal-related activity.

#### Single-cell analysis

We next investigated further whether value coding was completely absent from the place cell population by focusing on individual cells. We compared, in each condition (1vs0 or 1vs3) and for each goal (left or right) separately, the firing rate for that goal when value was changed to its firing rate in the preceding reference session. To do so, we computed the firing preference index between goals visited at least five times in the sessions of interest. The firing preference index was statistically tested against a shuffled distribution of the same data (see Materials and Methods). The results were evaluated in two ways. First, we labeled a cell as transiently value modulated if it had at least one instance of value coding, whether positively (higher firing in the value condition) or negatively (lower firing in the value condition). We found that 25% (*n* = 32) of place cells recorded in the 1vs3 condition were transiently value modulated, as were 17.5% (*n* = 29) of place cells recorded in the 1vs0 condition. When CA1 and CA3 place cells were considered separately, the proportions of transiently value-modulated cells were similar (in 1vs3, 24% for CA1, 26% for CA3; in 1vs0, 14% for CA1, 23.5% for CA3). Then, we considered that a true value-coding cell, as for a place cell, would have to consistently encode goal value. Therefore, we labeled cells as consistently value coding if they coded value in the same way (i.e., either by a significant increase or decrease of firing rate) for more than half of the instances in which this cell had been recorded in the same value condition. Twenty-nine repeatedly recorded place cells were analyzed either for 1vs3 (18 CA1 place cells, 11 CA3 place cells) or 1vs0 sessions (17 CA1, 12 CA3 place cells). None of the 29 place cells tested was consistently value coding in any of the conditions.

Therefore, in our task, place cells sometimes increased or decreased their firing following a change in goal value in individual sessions in similar proportions to what was previously reported in tasks with fewer spatial demands (in CA1, H Lee et al., 2012, in CA1 and CA3, 2017, Tryon et al., 2017). However, this could be due to global fluctuations of firing rate independent from goal value, because individual cells did not consistently encode the value of the goals. Overall, when individual and population results are taken together, place cells did not encode goal value in our paradigm.

### LFP: theta rhythm at the goal

**Figure 8.**
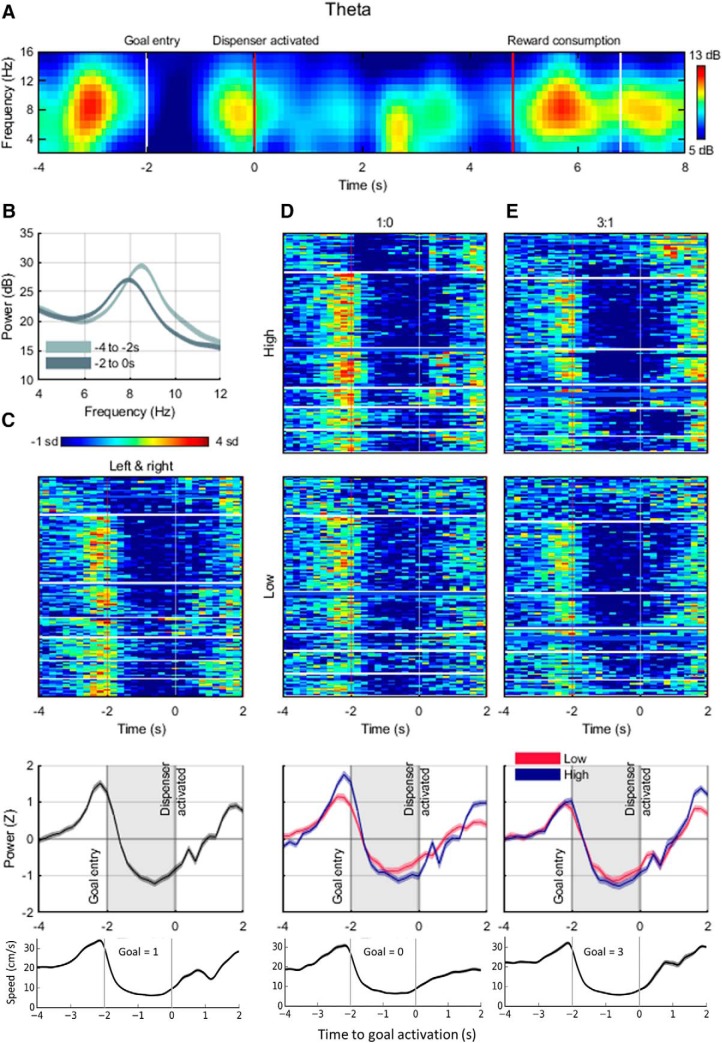
Theta power and frequency at the goal. ***A***, Individual example of power spectral density estimate in the theta band for 12 s of task behavior. Note the decrease in theta power during the goal period and the increase following reward consumption. ***B***, Average frequency over all 1vs1 sessions in the theta band before (−4:−2) and during (−2:0) the goal period. Note the decrease in frequency when waiting at the goal, as well as the decrease in power. ***C***, Top, Average *Z*-scored power in the theta band for each 1vs1 session around the goal period (left and right goals were combined because none of the parameters of interest was different between them). White horizontal lines indicate boundaries between different rats. Middle, Averaged *Z*-scored power in the theta band over all 1vs1 sessions. Note the decrease in theta power upon entry in the goal zone. Bottom, Averaged speed at the goal for these sessions accumulating left and right trials. Note the striking similarities between theta power and speed. ***D***, *Z*-scored theta power for each of 1vs0 sessions either for the high-value goal (1 pellet, top) or the low-value goal (0 pellet, middle). The bottom part shows the averaged *Z*-scored theta power, together with the average speed for the low-value goal. Note how theta power appears different between the two value conditions. ***E***, *Z*-scored theta power at the goal as for ***D*** but in 1vs3 sessions for the high-value goal (3 pellets, top) or the low-value goal (1 pellet, middle). Averaged *Z*-scored power and speed for the high-value goal are shown below. Note the similarity of theta power between the two value conditions.

Finally, we focused on hippocampal theta (4–12 Hz) and analyzed its power and frequency at the goal, first in the equal-value condition, and then in the modified-value conditions. An example of theta power density estimate is shown in Figure 8*A*. The left goal theta power (median = 25.5) did not differ from the right goal theta power (median = 25.3, Wilcoxon signed-rank test, *Z* = 0.01, *p* = 0.9) and the theta frequency at the left goal (median = 7.8 Hz) did not differ from that of the right goal (median = 7.9 Hz, Wilcoxon signed rank test, *Z* = −1.3, *p* = 0.19), so the two sides were combined. Figure 8*B* shows the power spectral density estimates just before goal zone entry (−4 to −2 s) and during the goal delay (−2 to 0 s) for the two goals combined and averaged over all 1vs1 sessions. The power and frequency of the theta rhythm decreased during the goal delay period (−4:−2 s), median power = 7.8, median frequency = 7.8 Hz, compared with pregoal period (−2:0 s), median power = 26.4, median frequency = 8.3 Hz, Wilcoxon on power, *Z* = 12.7, *p* = 2.4 × 10^−37^; Wilcoxon on frequency, *Z* = 9.3, *p* = 1.9 × 10^−20^). Therefore, theta power and frequency both decreased significantly in the goal zone compared with the previous 2 s, as was observed in the single-goal version of the task (Hok et al., 2007a).

Our paradigm allows the comparison of theta parameters for very similar behaviors when the rat is expecting different amounts of reward (cf. Fig. 8*D*,*E*). Comparisons of theta power between the different value conditions (whether for 1vs0 or 1vs3 sessions) did not show any significant difference (low vs high goal for 1vs0, Wilcoxon *Z* = −0.8, *p* = 0.40; low vs high goal for 1vs3, Wilcoxon *Z* = −0.51, *p* = 0.60). However, the theta frequency was significantly different between the low- and high-value goal, but only for 1vs0 sessions (low-value goal median = 7.9 Hz, high-value goal median = 7.7 Hz, Wilcoxon *Z* = 5.6, *p* = 1.9 × 10^−8^). For 1vs3 sessions, theta frequencies at the two goals were not different (Wilcoxon *Z* = 0.36, *p* = 0.72).

Overall, the results from the LFP analysis show correlates with running speed. However, there were no consistent differences evident as a function of expected or actual reward value even though, behaviorally, the rats distinguished the goal types. The only difference observed, between goals providing 1 or 0 pellets, might require more investigation, as a subtle difference in that condition was also observed in the hippocampal cells' PETHs profile. The only difference during the goal period between the 0 value condition and the others, aside from the difference in value, is that the next action of the rat should be to go to the other goal instead of exploring the arena for food. Perhaps rats in that condition are anticipating or planning a different type of action/trajectory and this might contribute to the subtle differences observed. Overall, our LFP results somewhat diverge from the recent findings of Tryon et al. (2017), who found effects of varying reward probability and other parameters such as agency on the theta rhythm; we suspect that these contrasting results arise from the differences in task demands.

## Discussion

We investigated whether hippocampal place cells are sensitive to goal value, consistent with a role for hippocampus in goal-directed navigation. We found that rats can learn to navigate to either of two unmarked goal locations and prefer the more rewarding goal. We replicated previous observations of out-of-field, goal-related activity from dorsal CA1 place cells (Hok et al., 2007a,b, 2013; Hayashi et al., 2016) and extended these findings to CA3. However, place cells did not encode goal value either with their place field or out-of-field firing. We conclude that during flexible navigation, dorsal hippocampal place cells encode space in a value-free manner. These findings and conclusions are examined in detail below.

### Electrophysiological markers of goal encoding

Rats were able to navigate efficiently to the goals and were also attuned to the 2 s interval between reaching the goal zone and reward delivery (or absence thereof), demonstrating knowledge of the goal locations. Electrophysiologically, we first looked for evidence of goal encoding because multiple studies have observed overrepresentation of goal locations by place fields (Hollup et al., 2001; Kobayashi et al., 2003; Lee et al., 2006; Dupret et al., 2010) or out-of-field firing (Hok et al., 2007a,b, 2013; Hayashi et al., 2016).

First, we did not see place fields clustered around the goals; this is similar to previous reports but stands in contrast to several other studies for reasons that appear task-related. Tasks that seem least likely to elicit goal overrepresentation involve variable behavioral phases (e.g., goal-directed alternating with foraging), dissociate goal from reward location, and/or involve multiple routes to the goal (Wiener et al., 1989; Speakman and O'Keefe, 1990; Markus et al., 1995; Gothard et al., 1996; Zinyuk et al., 2000; Jeffery et al., 2003; Anderson et al., 2006; Ainge et al., 2007, 2012; Hok et al., 2007a,b; Grieves et al., 2016; Spiers et al., 2018). In contrast, goal overrepresentation by place fields has been observed in tasks that entail the use of repeated, overlapping trajectories, provide reward at the goal location, and may include a novelty aspect (Eichenbaum et al., 1987; Breese et al., 1989; Kobayashi et al., 1997, 2003; Hollup et al., 2001; Fyhn et al., 2002; Lee et al., 2006, 2017; Dupret et al., 2010; H Lee et al., 2012; McKenzie et al., 2013; Mamad et al., 2017; Tryon et al., 2017; Gauthier and Tank, 2018). Further research is clearly needed to untangle the role of these parameters.

In contrast to the absence of place field clustering at goals, we found increased out-of-field population spiking at the goals occurring when the rat was paused or moving very slowly and developing after ∼1 s spent at the goal. This replicates previous findings from one-goal versions of the continuous navigation task (Hok et al., 2007a,b, 2013; Hayashi et al., 2016). Such activity has not been seen in tasks in which animals did not have to wait at the goal (Zinyuk et al., 2000; Anderson et al., 2006) or waited with a shorter delay (Siegel et al., 2008). Moreover, goal-firing activity was attenuated and occurred earlier in a cued version (Hok et al., 2007a). Finally, we found that this firing was independent of goal value, which suggests that the goal firing may relate to parameters that are more spatial than motivational; for example, a confirmation that the current location is indeed a goal.

Arguably, goal-related firing might be related to reactivation phenomena such as theta sequences (Foster and Wilson, 2007) or sharp-wave/ripple (SWR)-related replay (Foster and Wilson, 2006; Pfeiffer and Foster, 2013). Theta sequences, which are sequential place cell firing events occurring within one or a few theta cycles, generally during running, might reflect route planning because they have previously been found to extend toward goal locations (Wikenheiser and Redish, 2015). However, in our case, the rat was at the goal during the expression of goal firing and its future route was still unpredictable at this stage; therefore, theta sequences could not contain relevant future trajectory information. Firing during the goal-waiting period is thus unlikely to be attributable to theta sequences. Alternatively, SWRs are known to co-occur during replay of place cell sequences, usually during pauses around the reward location. We think our data are unlikely to reflect SWRs for several reasons. First, in our previous study (Hok et al., 2007a), no SWRs were observed during the goal zone periods. Second, in a circular track task, waiting for reward at a goal was linked to a decrease of SWRs (McKenzie et al., 2013). Finally, reduced goal firing in mutant mice was not found to be linked to reduced SWR activity at the goal (Hayashi et al., 2016). For these reasons, we did not attempt to optimize our setup for the artifact-free detection of SWRs. That said, we did look at the LFP data and did not see evidence of goal-related SWR activity (data not shown). Overall, it seems unlikely that goal-related firing could be an expression of SWR-related replay.

### Electrophysiological markers of goal value

Our main result is that although rats adapted their goal choices as a function of goal value, place cell activity was unaffected by changes in goal value. Place fields did not move toward the more (or less) rewarded locations, nor did they significantly change their pattern of firing as a function of goal value. The overdispersion of cell firing did not change between equal-value or value-changing sessions and was generally low, being similar to equivalent tasks with one goal of constant value. Goal firing did not increase at the low- or high-value goal and although a small proportion of individual cells transiently modulated their firing when goal value changed, this effect did not last over successive recordings of the same cell, in contrast to the stability of place cells' spatial firing.

Relatively few studies have addressed the issue of value coding in the hippocampus. In the majority of these studies, reward consumption co-occurred with the spatial location of the goal, making it difficult to differentiate goal from reward. One such study, in agreement with ours, found no evidence of goal value encoding (i.e., number of water drops delivered at a location; Tabuchi et al., 2003). Other reports suggest that place cells may encode reward probability, action value, or reward expectation signals in linear mazes that did not require to locate a spatially defined unmarked goal (H Lee et al., 2012; Lee et al., 2017; Tryon et al., 2017). Goal-value findings in these studies might instead relate to emotional enhancement of firing. For example, in a study by Tryon et al. (2017), rats had to choose between left and right doors on a forked linear maze, which provided either a low reward with 100% probability or a high reward with variable probability. Place cells fired more for the lowest probability (12.5%) choice, but only when reward was going to be delivered. One explanation for this could be that the occurrence of an unexpected reward triggers neurochemical reward signals (“surprised pleasure”). Our study, by contrast, mostly focused on the period before choice feedback was available, and dissociated goal and reward locations: in this case, no goal value encoding was found in the activity of place cells. Tryon et al. (2017) also found that reward probability and other task variables influenced theta power. In our paradigm, only trials in which the rat waited at the zero-pellet goal yielded subtle electrophysiological differences in the form of a blunted firing profile during the latter half of the delay or a lower theta frequency; these might also reflect emotional responses to the realization that reward would not follow. Finally, the hippocampus might encode goal value via modulations of place cell reactivations (either theta sequences or ripple-related replay; Ambrose et al., 2016) or other phenomena that the present study did not address (e.g., phase precession). Future studies will need to combine large-scale recordings with a spatial task to assess whether these parameters might encode the value of a spatial goal; such a result would be especially surprising given that individual place cells do not appear to encode goal value.

Overall, the idea that place cells are coding space independently from value is consistent with the theory of a predominantly spatial cognitive map (O'Keefe and Nadel, 1978). Neurons encoding goals, their value, or different decision-making parameters such as reward expectation, have been found in other brain regions (Doya, 2008). Reviewing these reports goes beyond the scope of the present study, so we will focus on two regions that receive inputs from the hippocampus: the prefrontal cortex and the striatum. Goal cells or reward-related signals were found in the medial prefrontal cortex (Hok et al., 2005) and in the orbitofrontal cortex (Feierstein et al., 2006; Riceberg and Shapiro, 2017); for reviews, see Wikenheiser and Schoenbaum (2016), Grieves and Jeffery (2017), and Poucet and Hok (2017). Additionally, the striatum is involved in reward processing, expected outcome coding, and possibly combining reward and place information (Lavoie and Mizumori, 1994; Lansink et al., 2009; Gmaz et al., 2018). It is thus likely that the hippocampus focuses on spatial encoding (in particular in a task requiring accurate spatial navigation), whereas other brain structures evaluate goal value and link this information with goal location.

### Conclusion

Our results suggest that, in an open-field arena with high navigation demands and low route stereotypy (high variability), place cells show evidence of goal-related activity but no evidence of goal value coding. What electrophysiological changes we did see may reflect neurochemical processing of reward expectation/anticipation, for which future studies of cellular excitability, as well as studies that assess the repeatability of the encoding of the phenomenon of interest, may be illuminating. Overall, our results support a predominantly spatial memory function for the rodent hippocampus and suggest that additional features of an environment, such as the value of specific places, are added to the “map” by downstream structures.
